# Screening anticancer activity by Brine shrimp lethality test of extracts of *Annona stenophylla* (Engl. & Diels), *Strophanthus petersianus* (Klotzsch) and *Synadenium glaucescens* (Pax)

**DOI:** 10.1371/journal.pone.0336636

**Published:** 2026-01-02

**Authors:** Roberto Luis Nhamussua, Faith Philemone Mabiki, Alinanuswe Joel Mwakalesi, Lyndy Joy McGaw

**Affiliations:** 1 Department of Natural Sciences, Faculty of Natural and Exact Sciences, Save University, Massinga, Inhambane, Mozambique; 2 Department of Chemistry and Physics, College of Natural and Applied Sciences, Sokoine University of Agriculture, Morogoro, Tanzania; 3 Phytomedicine Programme, Department of Paraclinical Sciences, Faculty of Veterinary Science, University of Pretoria, Pretoria, South Africa; Annamalai University, INDIA

## Abstract

Cancer continues to be one of the main public health challenges, driving the search for new compounds with therapeutic potential. Medicinal plants represent a valuable promising source of bioactive metabolites, and the Brine Shrimp Lethality Test has been widely used as a preliminary tool to assess the toxicity of natural extracts, providing clues to their possible anticancer activity. In this study, the cytotoxicity of the extracts of *Annona stenophylla* (Engl. & Diels), *Strophanthus petersianus* (Klotzsch), and *Synadenium glaucescens* (Pax) was investigated using the BSLT as a first step in screening for potential anticancer compounds. The plant materials were harvested in Tanzania and air-dried in the shade, and ground. The extracts were prepared by total sequential solvent extraction using cold maceration, starting with ethyl acetate, followed by methanol. A total of 24 ethyl acetate and methanolic extracts were obtained from the leaves, stem bark, stem wood, root wood and root bark of the three plants studied. The toxicity of the extracts was assessed by exposing *Artemia salina* nauplii to different concentrations of the extracts, with mortality recorded after 24 h. The LC_50_ was determined to evaluate the toxicity of each extract. All the extracts from the three plants exhibited different degrees of toxicity, with *A. stenophylla* demonstrating the lowest LC_50_ values, indicating the highest toxicity. The methanolic extract of *A. stenophylla*’s root wood exhibited the highest toxicity, producing a mortality rate of 99.44%, corresponding to an LC_50_ < 20 μg/mL. The observed toxicity suggests the presence of bioactive compounds with potential anticancer activities. The results support the potential of *A. stenophylla*, *S. petersianus* and *S. glaucescens* as sources of bioactive compounds with possible anticancer activity. Further studies, including phytochemical analysis and *in vitro* anticancer assays, are recommended to identify and characterize the active constituents responsible for the observed cytotoxic effects.

## Introduction

Despite advances in technological development and cancer treatment strategies, cancer continues to be an increasingly common disease and is considered one of the main causes of death worldwide [1]. It is characterized by uncontrolled cell growth that spreads abnormally throughout the body due to aberrations in numerous cell signaling circuits [2]. Different types of cancer are typically named according to the type of tissue or organ in which they originate [3]. The causes of most cancers are still unknown, nevertheless, factors such as lifestyle habits (e.g., smoking), excessive body weight, hormonal influences, and non-modifiable factors like genetic aberration are considered major contributors [3,4]. Almost every year, millions of new cases and deaths from cancer are recorded. According to the International Agency for Research on Cancer (IARC), in 2022, there were close to 20 million new cases of cancer and 9.7 million deaths, including non-melanoma skin cancer [5]. The same source reports that, based on projections from 2022 to 2050, the global cancer burden is expected to increase by 77%. Therefore, new strategies or compounds need to be discovered to provide effective treatment with fewer adverse effects because most cancer treatments, such as chemotherapy and radiotherapy, can cause fatigue, nausea, vomiting, hair loss, dizziness, lack of appetite and others, which in most cases, lead to the patient abandoning the treatment [6–8]. Medicinal plants have been used for years and are widely explored as a natural source of active compounds for treating cancer and other diseases. Of the total number of clinically approved anticancer drugs, it can be said that natural products contribute more than 25% [9]. For instance, vinblastine and vincristine are anticancer drugs isolated from *Catharanthus roseus* and clinically approved for cancer treatment [3].

Similarly, in the present study, anticancer screening was carried out using the Brine shrimp lethality test (BSLT) method of extracts of *Annona stenophylla*, *Strophanthus petersianus* and *Synadenium glaucescens* as the basis for the development of the species under study as raw material herbal medicine.

The plants were selected based on their use in folk medicine for the treatment of various diseases including cancer [10,11], and the fact that members of their families are well-known as sources of bioactive compounds with various pharmacological properties, including anticancer [12–14]. *A. stenophylla* belongs to the Annonaceae family, is known for its bioactive metabolites such as acetogenins, flavonoids and alkaloids with cytotoxicity and anticancer potential [15]. *S. petersianus* is a member of the Apocynaceae family and belongs to a group of plants traditionally used in African medicine to treat many diseases, including cancer. Furthermore, from *C. roseus,* another member of this family, were isolated vinblastine and vincristine, the first natural drugs used in cancer therapy and still being the most used in cancer treatment [16]. Some species of the *Strophanthus genus* are known to contain cardiac glycosides that may exhibit cytotoxicity effects [17,18]. Due to its bioactive compounds, *S. glaucescens,* from the Euphorbiaceae family, has been used in traditional medicine for various diseases, including cancer [19]. For instance, Euphol and Lupeol are the terpenes isolated from *S. grantii* and *glaucescens* [14,20,21], and β-sitosterol, a steroid isolated from diverse species including *S. glaucescens* [14,22,23], are anticancer agents and therefore candidates for cancer treatment. Further, Euphol revealed *in vitro* cytotoxicity against B16F10 melanoma cell lines, and an *in vivo* assay showed a significant reduction in tumour volume in melanoma-bearing mice [20], while Lupeol demonstrated anti-neoplastic effects against A549, a human non-small cell lung cancer cell line [21], and β-sitosterol exhibited cytotoxic activity against MCF-7 cancer cell lines [22].

*A. stenophylla* is commonly known as the dwarf custard apple in English, and is referred to as *Mtopetope* in Swahili, particularly in Tanzania [24,25]. The species has been recorded in Tanzania, Zambia, Zimbabwe, Angola, Botswana, Mozambique, the Republic Democratic of Congo and Namibia in woodland and sandy grassy slopes at the edge of wetlands [10,25]. In Tanzania, *A. stenophylla* can be found in Western, Rukwa, Tabora and Iringa Regions [25], and its fruits are particularly appreciated by herdsmen and children, who consume them for their naturally sweet, non-alcoholic juice [10]. The plant is commonly used in folk therapy as a snake repellent, for body swelling, managing diabetes, constipation, stomach pains, chest pain, blood purification, menorrhagia, dysmenorrhea, gonorrhoea, syphilis antiemetic and muscle sprains [26].

The species *S. petersianus,* commonly known as the sand forest poison rope, has been used as a poison for arrows and by the Zulus as an amulet against evil. It is native to countries from southern Kenya to South Africa [27].

*S. glaucescens* is commonly known as the milk bush plant in English and *Mvunjakongwa* in Swahili [28]. The plant is endemic in the East African Region and occurs in Tanzania, Kenya, the Democratic Republic of Congo and Burundi [29]. In Tanzania, it is distributed across diverse regions and is traditionally used for treating wounds, skin therapy, toothache, cough, tuberculosis, sexually transmitted infections, Human Immunodeficiency Virus (HIV), gastrointestinal worms and ringworms, excessive menstruation and asthma therapy [28,30–33].

The BSLT method is the first step for testing the toxicity of an extract or compound; it is also used to determine the bioactivity of a compound from a natural product. It is widely used for the pre-screening of active compounds in plant extracts and has a spectrum of pharmacological activity [34]. It is easy to perform, simple, fast and does not require a large cost with a 95% confidence level [35]. The BSLT method uses *A. salina* larvae as experimental invertebrate animals, where the toxicity of compounds is expressed by the LC_50_ value. The LC_50_ value indicates the concentration of compounds that causes the death of *A. salina* larvae in 50% of the population [36]. The method was applied in this study because it has a positive correlation with cytotoxicity tests using cancer cell culture. Therefore, it is often used as a tool for screening anticancer compounds [37].

Previous studies of *A. stenophylla* have assessed the antioxidant activity of its extract [38]. On another hand, researchers have investigated whether the root extract can inhibit α-glucosidase and α-amylase enzyme in the presence of carbohydrate substrates, suggesting a possible mechanism of its antidiabetic activity [39]. Additional studies include acute and subacute toxicity tests of its roots using rats, as well as, screening its roots and leaves by BSLT of [26,40]. No studies have been found regarding the species *S. petersianus*. Among the various studies conducted on *S. glaucescens*, Mabiki [30], assessed the toxicity of extracts from the root bark, root wood, stem bark, stem wood and leaves using the BSLT method. The findings suggested that these extracts possess potential anticancer properties.

Despite their use in folk medicine and the information outlined above, reports on screening for anticancer activity using the BSLT remain limited. This study aims to evaluate the potential anticancer activity of these plant extracts using the BSLT. The results provide preliminary insights into their toxicity, which may justify further investigations into their potential as sources of anticancer compounds.

## Materials and methods

### Plant collection and processing

The species were harvested in different locations according to their availability and abundance. The *A. stenophylla* (Engl. & Diels) and *S. petersianus* (Klotzsch) species were harvested from Pugu Forest Reserve in Dar es Salaam and Msubugwe Forest Reserve in Pangani district, respectively in February 2024. *S. glaucescens* (Pax) was collected in Mtumbatu, Tanzania, in October 2023. The botanical professional identified the plant species and registered the voucher specimens deposited for reference at the Institute of Traditional Medicine Herbarium (ITMH) with the numbers and coordinates as illustrated in Table 1.

**Table 1:** Voucher specimen numbers and coordinates of the species harvested.

Species	Voucher specimen	Coordinates
*A. stenophylla*	SH1502	UTM 0º5’05.632“ S and 9º2’33.735” E 203 m
*S. petersianus*	SH1501	UTM 0º4’68.350“ S and 9º3’89.255” E 109 m
*S. glaucescens*	SH1500	UTM 6º8’53.334“S and 36º59’21.615” E

The plants under study were collected using sustainable methods, which involved harvesting only the specific parts required for the research [41]. This approach aimed to minimise waste, prevent overharvesting, and ensure the long-term preservation of plant populations. All species were accurately identified by a qualified botanist, and representative samples were conserved for future research. Plant materials were manually harvested using appropriate tools such as pruning shears and small knives, depending on the plant part. Leaves were handpicked to avoid damaging the delicate structure; stems and bark were carefully cut using pruning shears. Similarly, roots were excavated with hoes and subsequently sectioned along with their bark using machetes. Fruits were not available at the time of collection. All materials were placed in breathable paper bags to avoid moisture retention during transport.

To avoid the decomposition of temperature light-sensitive compounds, all collected plant materials were air-dried under the shade (approximately 25 °C) in the Department of Chemistry and Physics laboratory at the Sokoine University of Agriculture. The plant materials were dried until they achieved a constant weight, determined over three consecutive days of weighing. The leaves of *A. stenophylla* and *S. petersianus* required 12 days to reach this point, whereas the leaves of *S. glaucescens,* due to their higher moisture content, required 23 days to reach a constant weight. The bark and wood of *A. stenophylla* and *S. glaucescens* were dried separately, and they took approximately 15 days to dry completely. For *S. petersianus,* the roots and stem were not separated from their bark and required 15 days to dry. The dried materials were ground using an electric mill (Silver Crest brand) to ensure uniform particle size, then weighed and packaged in polyethene zipper bags.

### Ethics statement

This study did not involve human participants or vertebrate animals and therefore did not require ethical approval. Field collection of plant material was conducted with permission from the Directorate of Postgraduate Studies, Research, Technology Transfer and Consultancy (DPRTC) of Sokoine University of Agriculture under reference number SUA/DPRTC/PYT/D/2022/0001/08. The study complied with all institutional, national, and international guidelines for the collection and use the plant materials in research.

### Extraction

Each harvested plant part (Table 2) was subjected to total and sequential solvent extraction using cold maceration, starting with ethyl acetate (EtOAc) to extract low to medium polarity compounds, followed by methanol (MeOH) to obtain more polar constituents [14,19].

**Table 2:** Harvested plant parts and corresponding masses used for extraction.

Species/parts	Root bark	Root wood	Stems bark	Stems wood	Leaves
*A. stenophylla*	1500 g	2400 g	1500 g		1900 g
*S. petersianus*	1400 g		2000 g		1700 g
*S. glaucescens*	2000 g	1600 g	1000 g	1600 g	1000 g

For this process, approximately 300 g or 400 g of each plant part (depending on its availability and density) was packed in amber bottles and extracted sequentially with 2 L of solvent, beginning with EtOAc and followed by MeOH. The mixture in the bottle was manually shaken for around 4 minutes and stored in a dark place to continue the extraction process. The extraction process for each solvent lasted for 72 h at room temperature, and each extraction was repeated three times, thereby ensuring maximum extraction [14]. The extracts were filtered using Whatman Nº 1 filter paper under gravity-assisted filtration. The resulting filtrate was then concentrated in a rotary evaporator (BUCHI), air-dried to evaporate the remaining solvent, placed in the desiccator to remove moisture, weighed and stored at – 20 °C for further analysis. A total of 24 crude extracts (EtOAc and MeOH) were obtained, their yield were calculated using the formula provided below and the yields values can be viewed within Table 3 of the results section.

**Table 3:** Percentage Yield of extracts and LC_50_ in BSLT *in vivo* study of extracts of *A. stenophylla, S. petersianus* and *S. glaucescens* vs toxicity classification according to Meyer and Clackson index.

Extracts tested	Extract (g)	% Yield (w/w)	Mortality values	Average number of dead larvae	Mortality (%)	LC_50_ (μg/mL)	Toxicity classification
Root bark of *A. stenophylla* EtOAc	41.09	2.74	170	9.44	94.44	< 20	High toxic
Root bark of *A. stenophylla* MeOH	87.82	5.85	86	4.90	47.78	193.86	Medium toxic
Root wood of *A. stenophylla* EtOAc	60.97	2.54	175	9.72	97.22	< 20	High toxic
Root wood of *A. stenophylla* MeOH	40.05	1.69	179	9.94	99.44	< 20	High toxic
Stems of *A. stenophylla* EtOAc	20.28	1.40	172	9.55	95.56	< 20	High toxic
Stems of *A. stenophylla* MeOH	90.08	6.00	172	9.55	95.56	< 20	High toxic
Leaves of *A. stenophylla* EtOAc	235.00	12.42	148	8.72	82.22	< 20	High toxic
Leaves of *A. stenophylla* MeOH	291.00	15.32	94	5.33	52.22	80.00	High toxic
Roots of *S. petersianus* EtOAc	24.64	1.76	55	2.83	30.56	360	Medium toxic
Roots of *S. petersianus* MeOH	68.68	3.43	32	1.28	17.78	360	Medium toxic
Stems of *S. petersianus* EtOAc	27.01	1.35	29	1.61	16.11	98.65	High toxic
Stems of *S. petersianus* MeOH	26.26	1.31	61	3.38	33.89	360	Medium toxic
Leaves of *S. petersianus* EtOAc	164.50	9.25	6	0.33	3.33	>360	–
Leaves of *S. petersianus* MeOH	40.88	2.30	44	2.44	24.44	330.23	Medium toxic
Root bark of *S. glaucescens* EtOAc	114.00	5.70	149	8.27	82.78	<20	High toxic
Root bark of *S. glaucescens* MeOH	114.00	12.00	101	5.61	56.11	47.95	High toxic
Root wood of *S. glaucescens* EtOAc	31.00	1.57	153	8.50	85.00	<20	High toxic
Root wood of *S. glaucescens* MeOH	26.43	1.32	162	9.00	90.00	<20	High toxic
Stem bark of *S. glaucescens* EtOAc	107.00	6.73	135	7.50	75.00	<20	High toxic
Stem bark of *S. glaucescens* MeOH	67.91	4.24	17	0.94	9.44	<360	–
Stem wood of *S. glaucescens* EtOAc	30.01	1.50	17	0.94	9.44	<360	–
Stem wood of *S. glaucescens* MeOH	37.27	1.86	82	4.56	45.56	135.67	Medium toxic
Leaves of *S. glaucescens* EtOAc	65.30	6.53	68	3.78	37.78	259.80	Medium toxic
Leaves of *S. glaucescens* MeOH	54.20	5.40	54	3.56	35.56	300	Medium toxic
Positive control (Leaves of *C. roseus* MeOH)	0.99	15.03	32	1.78	17.8	360	Medium toxic


$$Yield\;(\%)=\frac{Amount\;of\;dried\;crude\;extract}{Amount\;of\;dry\;sample\;used}x\text{100}$$

### Toxicity test with brine shrimp lethality test method

**Fig 1:**
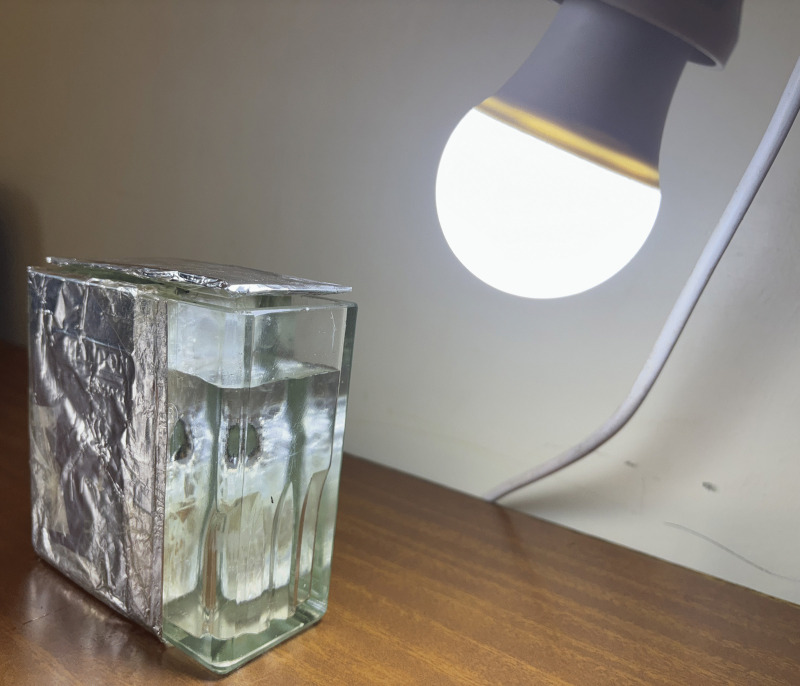
Hatching container for *Artemia salina* egg incubation.

The toxicity screening of all extracts was conducted using the BSLT method according to Credo et al. and Meyer et al. [19,42] with slight modifications. The brine shrimp eggs and artificial sea salt were supplied by the Laboratory of the Department of Chemistry and Physics, Sokoine University of Agriculture. The eggs were hatched in a rectangular container (22 x 32 x 6 cm) consisting of two unequal compartments connected by multiple small holes (Fig 1). Both compartments were filled with artificial seawater prepared by dissolving 3.8 g of crude artificial sea salt in 1 L of distilled water.

The crude artificial sea salt used was produced in the Laboratory by evaporating seawater collected from the Indian Ocean in Dar es Salaam, as detailed in (S1 File, Appendix 1).

Approximately 50 mg of eggs were spread in larger, darkened compartments. The smaller compartment was illuminated using a white LED lamp positioned approximately 10 cm above the water surface to stimulate phototactic movement and support larvae hatching. After 24 h, hatched *A. salina* larvae migrated toward the smaller, illuminated compartment due to their phototactic behaviour [42,43]. The larvae were collected from the lighted side, which was separated from the eggshells by a divider, after 48 h, using a 9-inch disposable pipette for use in the bioassay. The collection time allowed for complete hatching ensured adequate, viable, uniformly developed nauplii for the toxicity assay [43,44].

### Test sample preparation and implementation

Each EtOAc and MeOH extract obtained through sequential extraction was independently evaluated under the same assay conditions using the BSLT to determine their respective LC_50_ values. The bioassay was conducted according to the procedures described by Pohan et al. and Meyer et al. [34,42] with slight modifications. A total of 40 mg of each extract was accurately weighed, and dissolved in 1 mL of 1% dimethyl sulfoxide (DMSO) to prepare the stock solution. From this stock, a series of working solutions was prepared by dilution with artificial seawater to achieve final concentrations of 360, 240, 180, 80, 40, and 20 µg/mL, which were used to treat *A. salina* larvae.

Ten *A. salina* larvae 2 days old, were transferred into each well of a transparent, flat-bottomed 24-well plate (untreated), followed by the addition of 3 mL per well of the respective test sample. The experimental groups included the test samples, a negative control consisting of 1% DMSO in artificial seawater and a positive control, the methanolic leaf extract of *Catharanthus roseus.* The *A. salina* larvae in the experimental group were exposed to the test samples at the specified concentration. The control group received only 1% DMSO artificial seawater, while the positive control group was tested with *C. roseus* extract, known for its cytotoxic properties [45]. The experiment was conducted under consistent environmental conditions, such as a clean, well-ventilated area maintained at approximately 25 °C under continuous ambient light (~1000 lux), to simulate natural conditions and support the normal behaviour of *A. salina* larvae during the 24 h exposure period. Each concentration was tested in triplicate (n = 3 wells per concentration), providing a total of 30 larvae per concentration.

After 24 h of exposure, the number of live and dead larvae was recorded, and the mortality rate was calculated using the following equation. Larvae were considered dead if they showed no movement during observation under a magnifying lens, even after a gentle agitation.


$$\%\;death=\frac{number\;of\;dead\;nauplii}{number\;of\;the\;nauplii\;added\;in\;a\;vial\;}x\text{1}\text{0}\text{0}$$

### Statistical analysis

The median lethal concentration (LC_50_) values of *A. salina* (Brine shrimp) in both control and treatment groups were estimated using the four-parameter Hill equation [46].


Y=Bottom+ (Top − Bottom) (1 + (X / IC50)^ HillSlope

Where:

Y represents the percentage mortality

X represents concentration in μg/mL.

*Bottom* and *Top* represent the minimum and maximum response values, respectively, and *IC*_*50*_ corresponds to the concentration causing 50% mortality.

Non-linear least squares regression was performed using Python’s SciPy curve fit function with the Levenberg-Marquardt algorithm [47]. Model goodness-of-fit was assessed using the coefficient of determination (R^2^), calculated as:


$$R^{\text{2}}=\text{1}-\frac{{SS}_{res}}{{SS}_{tot}}+\ldots$$

Data handling and visualization were performed in Python 3 x using NumPy, SciPy, Pandas, and Matplotlib libraries.

Toxicity classification was based on established criteria. According to Meyer’s toxicity index, extracts with LC_50_ < 1000 μg/mL are considered toxic, while those with LC_50_ > 1000 μg/mL are considered non-toxic [42,48]. Clarkson’s toxicity index provides further organization:

LC_50_ > 1000 μg/mL → non-toxic

LC_50_ 500–1000 μg/mL → low toxic

LC_50_ 100–500 μg/mL → moderate toxicity

LC_50_ 0-100 μg/mL → high toxic [36,48].

## Results

**Fig 2:**
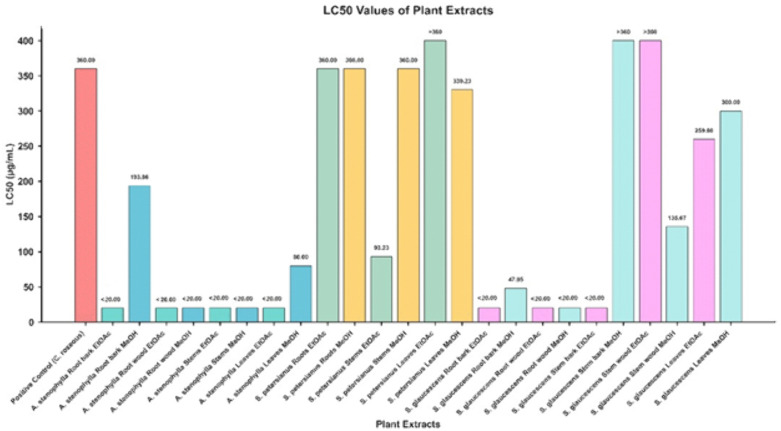
LC_50_ of the extracts compared with positive control.

The toxicity of plant extracts and the positive control was evaluated using the BSLT assay, and the corresponding LC_50_ values are summarised in Fig 2. As illustrated, substantial variation in LC_50_ values was observed among the different plant species, plant parts, and extraction solvents, reflecting distinct toxicity profiles.

The extracts of *A. stenophylla* consistently exhibited lower LC_50_ values, indicating high toxicity compared to both the control and other tested extracts. In contrast, *S. petersianus* displayed LC_50_ values comparable to the positive control, with the leaves EtOAc extract showing an LC_50_ > 360 μg/mL. Meanwhile, the roots, (bark and wood), stems bark (EtOAc extract), stem wood MeOH extract, and leaves (EtOAc and MeOH) of *S. glaucescens* demonstrated LC_50_ values which were lower than that of the positive control.

The percentage yield (w/w) of the extracts, mortality values including average, percentage of mortality of *A. salina* larvae, and toxicity classifications for all extracts tested are summarised in Table 3. The percentage yield represents the proportion of extract obtained relative to the total biomass initially subjected to extraction. Variation in extraction yield was observed across plant species, plant parts and solvents.

The mortality data revealed substantial variation in toxicity among the tested extracts. Across the concentration range of 360–20 μg/mL, LC_50_ values varied from less than 20 to higher than 360 μg/mL. High lethality against *A. salina* nauplii (LC_50_ < 100 μg/mL) was particularly evident in extracts derived from the roots, stems, and leaves of *A. stenophylla* in both solvents (EtOAc and MeOH), in the MeOH extract from the stems of *S. petersianus*, and the root bark and wood of *S. glaucescens.* The MeOH extract from the root wood of *A*. *stenophylla,* exhibited the highest toxicity, causing a 99.4% mortality rate in the *A. salina* [42,48]. In contrast, the MeOH extract from the root bark of *A. stenophylla*; the EtOAc and MeOH extracts from the roots; the EtOAc extract from the stems; and the EtOAc and MeOH extracts from the leaves of *S. petersianus*; as well as the EtOAc and MeOH extracts from the root bark and wood, the MeOH extract from the stem bark and stem wood, and the EtOAc and MeOH extracts from the leaves of *S. glaucescens* exhibited moderate toxicity (LC₅₀ 100–500 μg/mL). The EtOAc extract from leaves of *S. petersianus*, MeOH extract of root bark and EtOAc extract of stem wood from *S. glaucescens* exhibited the lowest toxicity, with an LC_50_ < 360 μg/mL [48]. Supporting data are provided in S1 File Tables 1–26. These results suggest that both the plant part and the extraction solvent significantly influence bioactivity.

The negative control (1% DMSO in artificial seawater) showed no mortality (0%) after 24 h, confirming that the solvent and experimental conditions did not affect the viability of *A. salina* larvae. In contrast, the positive control (MeOH extract of *C. roseus*), known for its cytotoxic activity, resulted in an LC_50_ of 360.00 μg/mL, thereby validating the sensitivity of the assay [49]. These control results confirm the reliability of the BSLT performed in this study.

## Discussion

Assessing the toxicity of plant extracts in cancer studies is crucial since many natural compounds have selective cytotoxic potential against tumor cells [50]. However, it is essential to determine their safety for healthy cells to develop effective therapies with minimal adverse effects [51]. This study searches for sources of potential antitumor agents through general toxicity, contributing to the development of new therapeutic agents. The BSLT method was used for screening the toxicity of the extracts from the species under study because this method has been shown to correlate positively with cytotoxic effect on cancer cells [52]. It is commonly employed as a pre-screening test to determine the mortality rate, which are directly proportional to the concentration of the extracts in drug discovery studies [53].

The results of this study are summarised in Table 3 of the results section. Thus, at the concentrations tested, the results demonstrate a wide range of toxicity among the plant extracts. The extracts of *A. stenophylla* and *S. glaucescens* exhibited relatively low LC_50_ values (LC_50_ < 100 μg/mL), indicating strong cytotoxic activity [48]. These findings suggest the presence of potent bioactive compounds with various pharmacological properties, including potential anticancer activity [36,54]. Although specific compound confirmation was not conducted in this study, the observed cytotoxic profiles are consistent with previous reports linking such activity to secondary metabolites, phenolic compounds, alkaloids, terpenoids, acetogenins, terpenes, tannins and other secondary metabolites in related studies [55,56]. Therefore, these extracts represent priority candidates for bioassay-guided fractionation, phytochemical profiling and targeted isolation of active components. In contrast, several other extracts exhibited moderate toxicity (LC_50_ > 100 < 500 μg/mL), which could contain lower-abundance active constituents that require concentration or enrichment [57,58]. The EtOAc extract from leaves of *S. petersianus,* MeOH extract of stem bark and EtOAc extract of stem wood from *S. glaucescens* displayed low toxicity (LC_50_ < 360 μg/mL) and are unlikely to be the major source of cytotoxic principles under the extraction conditions used [59].

Among the extracts screened, the methanolic extract from the root wood of *A. stenophylla* exhibited the highest toxicity, as the lowest concentration tested (20 μg/mL) caused 96.7% mortality in *A. salina* larvae (S1 File, Table 4). The pronounced toxicity of this species may be attributed to its diverse biological activities, including antioxidants of *A. stenophylla* reported by Maroyi [10], and the presence of polyphenolic compounds such as flavonoids [38]. This is further supported by the exceptionally low LC_50_ values less than 20 μg/mL observed for both the methanolic extracts root wood and root bark of *A. stenophylla* [48].

In general, at the tested concentrations (360, 240, 180, 80, 40 and 20 μg/ml), the EtOAc extracts demonstrated higher toxicity against *A. salina* larvae, as indicated by their greater average mortality rates and lower LC_50_ values (Table 3). In contrast, extracts from *S. petersianus* exhibited slightly higher LC_50_ values compared to those of the other two plant species.

This study incorporated both positive and negative controls to validate the cytotoxic assay. The methanolic leaf extracts of *C. roseus* (positive control) exhibited an LC_50_ value of 360.00 μg/mL. Based on LC_50_ values obtained in this study, together with previous reports by Meyer et al. and Kalauni et al. [42,49], several of the tested plant extracts were classified as pharmacologically active, with activities suggestive of potential anticancer properties when compared to the positive control. The negative control (1%DMSO) exhibited no significant lethality, thereby confirming the validity of the assay and the specificity of the cytotoxic response to the plant extracts. The use of these controls ensured the reliability of the assay system and allowed for a clean distinction between true cytotoxic effects and non-specific responses to the solvent.

According to Clarkson’s criteria, the tested extracts can be categorised as highly, moderately, and low toxic [48]. Furthermore, the American National Cancer Institute (NCI), considers extracts with an LC_50_ ≤ 30 μg/mL against cancer cells as promising candidates for purification and further development as anticancer agents [60,61]. In this study, certain plants parts exhibited notable cytotoxic activity based on their LC_50_ values, suggesting the presence of bioactive constituents that warrant further isolation, characterization and evaluation for potential anticancer applications [62,63]. Previous studies have also reported anticancer properties in other species belonging to the families Annonaceae, Apocynaceae and Ephorbiaceae, as well as in the *genera Annona* and *Synadenium* [15,20,64].

The bioassay with extracts of *S. petersianus* revealed high LC_50_ values; therefore, its extracts are considered medium and low toxic according to the Meyer and Clackson criteria index [42,48]. This might be caused by a lower concentration of bioactive constituents, including anticancer agents [57,65]. Although scientific information on *S. petersianus* remains limited, a few studies have documented its traditional use as an arrow poison by several African communities, including Zulu, attributed to its content of potent cardiac glycosides. Additionally, it has been employed in ritualistic and protective practices aimed and averting harm [66]. However, its cytotoxic and pharmacological properties, as well as its phytochemical profile, remain largely unexplored. This study contributes to addressing this knowledge gap by demonstrating the cytotoxic effects of *S. petersianus* extracts, suggesting its potential as a promising candidate for future anticancer research. Notably, other species within the *Strophanthus genus* have been reported to exhibit antimicrobial, wound-healing, antioxidant, analgesic, and anticarcinogenic properties [67,68].

The cytotoxic effects observed in this study are consistent with those by Mabiki [30], who investigated the toxic effects of *S. glaucescens* root bark and wood, stem bark, stem wood and leaves using various solvent extracts. In that study, the dichloromethane extracts exhibited the highest toxicity against *A. salina* nauplii, followed by petroleum ether and ethanol extracts. Although different solvents were used in the present study, the observed biological activity is in agreement with Mabiki’s findings [30], suggesting that key bioactive compounds are present across various plant parts and remain extractable by solvents of differing polarity.

Other studies, Yang et al., Credo et al. & Babu et al. [17,19,21], involving isolation of pure compounds from *S. glaucescens* have identified several classes of secondary metabolites, including terpenes, terpenoids, steroids and hydrolysable tannins, many of which are known to possess potential antitumor activity. The consistency between these reports and the current findings strengthens the hypothesis that *S. glaucescens* contains cytotoxic constituents of pharmacological relevance. This also highlights the need for further phytochemical characterisation and bioassay-guided fractionation of *A. stenophylla, S. petersianus,* and *S. glaucescens* to isolate and identify the active principles responsible for the observed cytotoxic effects [62].

However, some limitations remain. The relatively small sample size of the collected plant material may limit the generalizability of the findings. Additionally, although standard cytotoxicity methods were applied, future studies should incorporate mechanistic assays such as apoptosis or oxidative stress markers and include a broader set of biological models to better understand the mode of action and enhance the translational relevance of the results.

## Conclusion

This study demonstrated that the extracts of *A*. *stenophylla*, *S. petersianus* and *S*. *glaucescens* exhibit significant cytotoxic activity, supporting their traditional medicinal use and validating our hypothesis that these plants contain bioactive compounds with potential anticancer properties. Among the tested extracts, the methanolic extract from the root wood of *A. stenophylla* exhibited the highest toxicity, indicating the presence of potent cytotoxic constituents extractable by this solvent.

Although the use of *C. roseus* as a positive control and 1% DMSO as a negative control ensured the reliability of the results, several limitations must be acknowledged. This includes a limited sample size of the plant materials, a limited number of test organisms per well, a lack of detailed mechanistic studies, for instance, apoptosis, oxidative stress, and the absence of *in vivo* studies for validation. These factors may constrain the broader applicability of the findings.

Nonetheless, contribute to ongoing efforts to discover new anticancer agents with fewer adverse effects, as highlighted in the introduction. The observed cytotoxic provides preliminary evidence that this species could serve as a promising source of lead compounds. Future studies should focus on bioassay-guided isolation of active constituents, detailed phytochemical profiling, mechanistic studies and evaluation in more complex biological models to further assess efficacy and safety.

In light of the increasing global burden of cancer and the urgent need for safer, more effective treatments, this study represents a step forward in the scientific exploration of traditional medicinal plants as potential contributors to cancer drug discovery.

## Supporting information


S1 File.
**Appendix 1.** Detailed description of the method used to prepare artificial sea salt from seawater collected from the Indian Ocean. **Tables 1–26**. Raw data on the survival of *Artemia salina* larvae after 24 hours of exposure to different concentrations of extracts and control treatments.
(DOCX)

## References

[1] Orozco-Barocio A, Robles-Rodríguez BS, Camacho-Corona MDR, Méndez-López LF, Godínez-Rubí M, Peregrina-Sandoval J, et al. In vitro Anticancer Activity of the Polar Fraction From the Lophocereus schottii Ethanolic Extract. Front Pharmacol. 2022;13:820381. doi: 10.3389/fphar.2022.820381 35444555 PMC9014087

[2] Zainal Baharum A, Md Akim T, Yap Yun Hin RK. No title. Trop Life Sci Res. 2016;27:21–42. 27019680 PMC4807961

[3] Ukwubile CA, Ikpefan EO, Malgwi TS, Bababe AB, Odugu JA, Angyu AN, et al. Cytotoxic effects of new bioactive compounds isolated from a Nigerian anticancer plant Melastomastrum capitatum Fern. leaf extract. Scientific African. 2020;8:e00421. doi: 10.1016/j.sciaf.2020.e00421

[4] Pinheiro PS, Callahan KE, Jones PD, Morris C, Ransdell JM, Kwon D, et al. Liver cancer: A leading cause of cancer death in the United States and the role of the 1945-1965 birth cohort by ethnicity. JHEP Rep. 2019;1(3):162–9. doi: 10.1016/j.jhepr.2019.05.008 32039366 PMC7001577

[5] Bray F, Laversanne M, Sung H, Ferlay J, Siegel RL, Soerjomataram I, et al. Global cancer statistics 2022: GLOBOCAN estimates of incidence and mortality worldwide for 36 cancers in 185 countries. CA Cancer J Clin. 2024;74(3):229–63. doi: 10.3322/caac.21834 38572751

[6] Abdulridha MK, Al-Marzoqi AH, Al-Awsi GRL, Mubarak SMH, Heidarifard M, Ghasemian A. Anticancer Effects of Herbal Medicine Compounds and Novel Formulations: a Literature Review. J Gastrointest Cancer. 2020;51(3):765–73. doi: 10.1007/s12029-020-00385-0 32140897

[7] Huang M, Lu J-J, Ding J. Natural Products in Cancer Therapy: Past, Present and Future. Nat Prod Bioprospect. 2021;11(1):5–13. doi: 10.1007/s13659-020-00293-7 33389713 PMC7933288

[8] Brianna, Lee SH. Chemotherapy: how to reduce its adverse effects while maintaining the potency?. Med Oncol. 2023;40(3):88. doi: 10.1007/s12032-023-01954-6 36735206

[9] Newman DJ, Cragg GM. Natural Products as Sources of New Drugs over the Nearly Four Decades from 01/1981 to 09/2019. J Nat Prod. 2020;83(3):770–803. doi: 10.1021/acs.jnatprod.9b01285 32162523

[10] Maroyi A. Annona stenophylla Engl. & Diels: review of its botany, medicinal uses and biological activities. J Pharm Sci Res. 2019;11:3385–90.

[11] Maroyi A. An ethnobotanical survey of medicinal plants used by the people in Nhema communal area, Zimbabwe. J Ethnopharmacol. 2011;136(2):347–54. doi: 10.1016/j.jep.2011.05.003 21575701

[12] Amala Dev AR, Joseph SM. Anticancer potential of Annona genus: A detailed review. Journal of the Indian Chemical Society. 2021;98(12):100231. doi: 10.1016/j.jics.2021.100231

[13] Islam MS, Lucky RA. A study on different plants of apocynaceae family and their medicinal uses. Univ J Pharm Res. 2019. doi: 10.22270/ujpr.v4i1.235

[14] Rwegoshora F, Mabiki F, Machumi F, Chacha M, Styrishave B, Cornett C. Isolation and toxicity evaluation of feruloyl ester and other triterpenoids from Synadenium glaucescens Pax. J Phytopharmacol. 2022;11(5):347–52. doi: 10.31254/phyto.2022.11506

[15] Tundis R, Xiao J, Loizzo MR. Annona species (Annonaceae): a rich source of potential antitumor agents?. Ann N Y Acad Sci. 2017;1398(1):30–6. doi: 10.1111/nyas.13339 28415154

[16] Costa MMR, Hilliou F, Duarte P, Pereira LG, Almeida I, Leech M, et al. Molecular cloning and characterization of a vacuolar class III peroxidase involved in the metabolism of anticancer alkaloids in Catharanthus roseus. Plant Physiol. 2008;146(2):403–17. doi: 10.1104/pp.107.107060 18065566 PMC2245823

[17] Yang H-Y, Chen Y-X, Luo S, He Y-L, Feng W-J, Sun Y, et al. Cardiac glycosides from Digitalis lanata and their cytotoxic activities. RSC Adv. 2022;12(36):23240–51. doi: 10.1039/d2ra04464a 36090389 PMC9380703

[18] Wen S, Chen Y, Lu Y, Wang Y, Ding L, Jiang M. Cardenolides from the Apocynaceae family and their anticancer activity. Fitoterapia. 2016;112:74–84. doi: 10.1016/j.fitote.2016.04.023 27167183

[19] Credo D, Mabiki FP, Machumi F, Cornett C. Structural Elucidation and Toxicity Evaluation of Bioactive Compounds from the Leaves and Stem woods of Synadenium glaucescens Pax. Pharm Sci Res. 2022;9:59–66. doi: 10.7454/psr.v9i2.1262

[20] de Oliveira TL, Munhoz ACM, Lemes BM, Minozzo BR, Nepel A, Barison A, et al. Antitumoural effect of Synadenium grantii Hook f. (Euphorbiaceae) latex. J Ethnopharmacol. 2013;150(1):263–9. doi: 10.1016/j.jep.2013.08.033 24008110

[21] Babu TS, Michael BP, Jerard C, Vijayakumar N. Study on the anti metastatic and anticancer activity of Triterpene compound Lupeol in human lung cancer. Int J Pharm Sci Res. 2019;10:721–727. doi: 10.13040/IJPSR.0975-8232.10(2).721-27

[22] Li L, Zou Q, Chunduru J, Ibrahim MAA, Hassan EM, Laroe N, et al. Anti-tumor metabolites from Synadenium grantii Hook F. Med Chem Res. 2022;31(4):666–73. doi: 10.1007/s00044-022-02867-1

[23] Chang F, Chen C, Hsieh T, Cho C, Wu Y. Chemical Constituents from Annona Glabra III. J Chinese Chemical Soc. 2000;47(4B):913–20. doi: 10.1002/jccs.200000124

[24] Taderera T, Chagonda LS, Gomo E, Katerere D, Shai LJ. Annona stenophylla aqueous extract stimulate glucose uptake in established C2Cl2 muscle cell lines. Afr Health Sci. 2019;19(2):2219–29. doi: 10.4314/ahs.v19i2.47 31656507 PMC6794551

[25] Ruffo CK. Edible Wild Plants of Tanzania. 2002. https://agris.fao.org/search/en/providers/122621/records/647396b768b4c299a3fb6a61

[26] Chagonda LS, Tafadzwa M, Dexter T, Louis G, Exnevia G, Felicity B, et al. Acute and Sub-acute Oral Toxicity of Hydroethanolic Root Extract ofAnnona stenophyllaEngl. and Diels in Sprague Dawley Rats. Journal of Biologically Active Products from Nature. 2015;5(5):349–56. doi: 10.1080/22311866.2015.1110501

[27] Bester SP. Strophanthus amboensis. Flower Plants Africa. 2019;66:102–14.

[28] Msengwa Z, Rwegoshora F, David C, Mwesongo J, Mafuru M, Mabiki FP. Epifriedelanol is the key compound to antibacterial effects of extracts of Synadenium glaucescens (Pax) against medically important bacteria. Front Trop Dis. 2023;3:1–10. doi: 10.3389/fitd.2022.110454

[29] Nyigo V, Mdegela R, Mabiki F, Malebo H. Assessment of Dermal Irritation and Acute Toxicity Potential of Extracts from Synadenium glaucescens on Healthy Rabbits, Wistar Albino Rats and Albino Mice. EJMP. 2015;10(4):1–11. doi: 10.9734/ejmp/2015/20763

[30] Mabiki FP. Bioactivity potential of extracts from Synadenium glaucescens Pax (Euphorbiaceae). Sokoine University of Agriculture. 2013.

[31] Mabiki FP, Magadula JJ, Mdegela RH, Mosha RD. Optimization of Extraction Conditions and Phytochemical Screening of Root Extract of Synadenium glaucescens Pax. IJC. 2013;5(4). doi: 10.5539/ijc.v5n4p103

[32] Mabiki FP, Mdegela RH, Mosha RD, Magadula JJ. In ovo antiviral activity of Synadenium glaucescens (Pax) crude extracts on Newcastle disease virus. J Med Plants Res. 2013;7:863–70. doi: 10.5897/JMPR12.684

[33] Mabiki FP, Madege R. Natural occurrence of moulds and mycotoxins in Synadenium glaucescens extracts (SGE) under different storage conditions. Tanzania J Agric Sci. 2022;21:161–74.

[34] Pohan DJ, Marantuan RS, Djojosaputro M. Toxicity Test of Strong Drug Using the BSLT (Brine Shrimp Lethality Test) Method. Int J Health Sci Res. 2023;13(2):203–9. doi: 10.52403/ijhsr.20230228

[35] Rasyid MI, Yuliani H, Triandita N, Angraeni L, Anggriawin M. Toxicity Test of Laban Fruits (Vitex pinnata Linn) by Using Brine Shrimp Lethality Test (BSLT) Methode. IOP Conf Ser: Earth Environ Sci. 2022;1059(1):012051. doi: 10.1088/1755-1315/1059/1/012051

[36] Hamidi МR, Jovanova B, Panovska ТK. Toxic оlogical evaluation of the plant products using brine shrimp (Artemia salina L.) model. Maced Pharm Bull. 2014;60:9–18.

[37] Rachutami I, Martha RD, Muadifah A, Manggara AB. Anti-Cancer Activity Testing of Cumin (Plectranthus Amboinicus) Ethanol Extract Against Artemia Salina Leach by Using Brine Shrimp Lethality Test (BSLT) Method. Walisongo J Chem. 2022;5(1):19–28. doi: 10.21580/wjc.v5i1.9086

[38] Cosmas JM, Rudo FM. In vitro evaluation of fruit extracts of Annona stenophylla diels and Flacourtia indica for incorporation into formulations for management of cancer. J Med Plants Res. 2019;13(11):242–51. doi: 10.5897/jmpr2019.6746

[39] Taderera T, Taderera T, Gomo E. Inhibitory activity of α-glucosidase and α-amylase by Annona stenophylla root extract as mechanism for hypoglycaemic control of DM. Int J Pharm. 2015;106:436–44.

[40] Munodawafa. T. Screening of some traditional medicinal plants from Zimbabwe for biological and anti-microbial activity. Citeseer. 2005.

[41] Rokaya MB, Münzbergová Z, Dostálek T. Sustainable harvesting strategy of medicinal plant species in Nepal – results of a six-year study. Folia Geobot. 2017;52(2):239–52. doi: 10.1007/s12224-017-9287-y

[42] Meyer BN, Ferrigni NR, Putnam JE, Jacobsen LB, Nichols DE, McLaughlin JL. Brine shrimp: a convenient general bioassay for active plant constituents. Planta Med. 1982;45(5):31–4. doi: 10.1055/s-2007-971236 17396775

[43] El-Magsodi MO, Bossier P, Sorgeloos P, Van Stappen G. Effect of Light Colour, Timing, and Duration of Light Exposure on the Hatchability of Artemia Spp. (Branchiopoda: Anostraca) Eggs. Journal of Crustacean Biology. 2016;36(4):515–24. doi: 10.1163/1937240x-00002454

[44] Wulandari DD, Risthanti RR, Sari EAP, Anisa H, Filia S. Phytochemical screening and toxicological evaluation using Brine Shrimp Lethality Tes (BSLT) of ethanolic extract of Morinda citrifolia L. Bali Med J. 2022;11(2):561–5. doi: 10.15562/bmj.v11i2.3119

[45] Pham HNT, Vuong QV, Bowyer MC, Scarlett CJ. Phytochemicals Derived from Catharanthus roseus and Their Health Benefits. Technologies. 2020;8(4):80. doi: 10.3390/technologies8040080

[46] Weiss JN. The Hill equation revisited: uses and misuses. The FASEB Journal. 1997;11(11):835–41. doi: 10.1096/fasebj.11.11.9285481 9285481

[47] Sebaugh JL. Guidelines for accurate EC50/IC50 estimation. Pharm Stat. 2011;10(2):128–34. doi: 10.1002/pst.426 22328315

[48] Alim MA, Zaman NR, Hossain MN. Investigation of phytochemical properties of the methanolic extract of rosenvingea spp. found in the north-eastern region of the Bay of Bengal. Bioresearch Communications. 2022;9(1):1252–62. doi: 10.3329/brc.v9i1.63606

[49] Kalauni SK, Mahato SK, Khanal LN. Phytochemical Studies and Toxicity Evaluation of Selected Medicinal Plants from Sarlahi District, Nepal. JPlReso. 2023;21(1):21–31. doi: 10.3126/bdpr.v21i1.57199

[50] Solowey E, Lichtenstein M, Sallon S, Paavilainen H, Solowey E, Lorberboum-Galski H. Evaluating medicinal plants for anticancer activity. ScientificWorldJournal. 2014;2014:721402. doi: 10.1155/2014/721402 25478599 PMC4248331

[51] Calderón-Montaño JM, Martínez-Sánchez SM, Jiménez-González V, Burgos-Morón E, Guillén-Mancina E, Jiménez-Alonso JJ, et al. Screening for Selective Anticancer Activity of 65 Extracts of Plants Collected in Western Andalusia, Spain. Plants (Basel). 2021;10(10):2193. doi: 10.3390/plants10102193 34686002 PMC8537044

[52] H H, Irawan C, Sirait SM, Sulistiawaty L, Setyawati SR. Toxicity Test with BSLT (Brine Shrimp Lethality Test) Method on Methanol, Ethyl Acetate Extract, Hexane on Seeds and Rind of Matoa extract (Pometia pinnata). Orient J Chem. 2020;36(6):1143–7. doi: 10.13005/ojc/360618

[53] Rasyid MI, Yuliani H, Angraeni L. Toxicity Test LC50 of Pineung Nyen Teusalee Seeds (Areca catechu) Extract by Brine Shrimp Lethality Test (BSLT) Methode. IOP Conf Ser: Earth Environ Sci. 2020;515(1):012052. doi: 10.1088/1755-1315/515/1/012052

[54] Aksono EB, Latifah AC, Suwanti LT, Haq KU, Pertiwi H. Clove Flower Extract (Syzygium aromaticum) Has Anticancer Potential Effect Analyzed by Molecular Docking and Brine Shrimp Lethality Test (BSLT). Vet Med Int. 2022;2022:5113742. doi: 10.1155/2022/5113742 36106174 PMC9467815

[55] Bharadwaj R, Haloi J, Medhi S. Topical delivery of methanolic root extract of Annona reticulata against skin cancer. South African Journal of Botany. 2019;124:484–93. doi: 10.1016/j.sajb.2019.06.006

[56] Verengai W, Chitindingu K, Marume A, Pharmaceuticals G. An anti-diabetic poly-herbal medicine prepared from extracts of Annona stenophylla, Citrus limon and Zingiber officinales. 2017;8. doi: 10.13040/IJPSR.0975-8232.8(3).1048-55

[57] Clemen-Pascual LM, Macahig RAS, Rojas NRL. Comparative toxicity, phytochemistry, and use of 53 Philippine medicinal plants. Toxicol Rep. 2021;9:22–35. doi: 10.1016/j.toxrep.2021.12.002 34976744 PMC8685920

[58] Martins de Deus B, Fernandes C, Molina AK, Xavier V, Pires TCSP, Mandim F, et al. Chemical Characterization, Bioactivity and Toxicity of European Flora Plant Extracts in Search for Potential Natural Origin Preservatives. Plants (Basel). 2023;12(15):2784. doi: 10.3390/plants12152784 37570937 PMC10420968

[59] Assay AS. Phytochemical, anti-nutritional and toxicity assessment of Moringa. Jordan J Chem. 2018;13:171–8.

[60] Albuntana A, Yasman Y, Wardhana W. Toxicity test of extracts of the four sea cucumber (family holothuriidae) from east penjaliran island, seribu islands, jakarta based on the brine shrimp lethality test (bslt). J Ilmu dan Teknologi Kelautan Tropis. 2011;3(1). doi: 10.29244/jitkt.v3i1.7835

[61] Simorangkir M, Nainggolan B, Juwitaningsih T, Silaban S. The Toxicity of n-Hexane, Ethyl Acetate and Ethanol Extracts of SarangBanua (Clerodendrumfragrans Vent Willd) Leaves by Brine Shrimp Lethality Test (BSLT) Method. J Phys: Conf Ser. 2021;1811(1):012053. doi: 10.1088/1742-6596/1811/1/012053

[62] Canga I, Vita P, Oliveira AI, Castro MÁ, Pinho C. In Vitro Cytotoxic Activity of African Plants: A Review. Molecules. 2022;27(15):4989. doi: 10.3390/molecules27154989 35956938 PMC9370645

[63] Ali JS, Riaz N, Mannan A, Tabassum S, Zia M. Antioxidative-, Antimicrobial-, Enzyme Inhibition-, and Cytotoxicity-Based Fractionation and Isolation of Active Components from Monotheca buxifolia (Falc.) A. DC. Stem Extracts. ACS Omega. 2022;7(4):3407–23. doi: 10.1021/acsomega.1c05647 35128250 PMC8811920

[64] Perrone A, Yousefi S, Salami A, Papini A, Martinelli F. Botanical, genetic, phytochemical and pharmaceutical aspects of Annona cherimola Mill. Scientia Horticulturae. 2022;296:110896. doi: 10.1016/j.scienta.2022.110896

[65] Mailu JK, Nguta JM, Mbaria JM, Okumu MO. Qualitative and quantitative phytochemical composition, antimicrobial activity, and brine shrimp cytotoxicity of different solvent extracts of Acanthus polystachyus, Keetia gueinzii, and Rhynchosia elegans. Futur J Pharm Sci. 2021;7(1). doi: 10.1186/s43094-021-00342-z

[66] Magwede K, van Wyk B-E, van Wyk AE. An inventory of Vhavenḓa useful plants. South African Journal of Botany. 2019;122:57–89. doi: 10.1016/j.sajb.2017.12.013

[67] Wen S, Chen Y, Lu Y, Wang Y, Ding L, Jiang M. Cardenolides from the Apocynaceae family and their anticancer activity. Fitoterapia. 2016;112:74–84. doi: 10.1016/j.fitote.2016.04.023 27167183

[68] Bhalla A, Thirumalaikolundusubramanian P, Fung J, Cordero-Schmidt G, Soghoian S, Sikka VK, et al. Native Medicines and Cardiovascular Toxicity. Heart and Toxins. Elsevier. 2015. 175–202. doi: 10.1016/b978-0-12-416595-3.00006-2

