# Supplementary material for: Screening anticancer activity by Brine shrimp lethality test of extracts of Annona stenophylla (Engl. & Diels), Strophanthus petersianus (Klotzsch) and Synadenium glaucescens (Pax)
Source: PLoS One. 2026 Jan 2;21(1):e0336636. doi: 10.1371/journal.pone.0336636 (PMC12758728; doi:10.1371/journal.pone.0336636)
Supplement: S1 File. — Appendix 1. Detailed description of the method used to prepare artificial sea salt from seawater collected from the Indian Ocean. Tables 1–26. Raw data on the survival of Artemia salina larvae after 24 hours of exposure to different concentrations of extracts and control treatments. (DOCX) [file pone.0336636.s001.docx]

**Screening anticancer activity by Brine shrimp lethality test of extracts of *Annona* *stenophylla* (Engl. & Diels), *Strophanthus* *petersianus* (Klotzsch) and *Synadenium* *glaucescens* (Pax)**

Roberto Luis Nhamussua^1*^, Faith Philemone Mabiki^2¶^, Alinanuswe Joel Mwakalesi^2¶^ and Lyndy Joy McGaw^3¶^

^1^Department of Chemistry and Physics, College of Natural and Applied Sciences, Sokoine University of Agriculture, P.O. BOX 3038 Morogoro, Tanzania; and Department of Natural Sciences, Faculty of Natural and Exact Sciences, Save University, CP. 111 – Massinga, Inhambane, Mozambique.

^2¶^Department of Chemistry and Physics, College of Natural and Applied Sciences, Sokoine University of Agriculture, P.O. BOX 3038 Morogoro, Tanzania.

^3¶^Phytomedicine Programme, Department of Paraclinical Sciences, Faculty of Veterinary Science, University of Pretoria, Private Bag X04, Onderstepoort, 0110 Pretoria, South Africa.

*Corresponding author

E-mail: [rluisnha@gmail.com](mailto:rluisnha@gmail.com) (RLN)

# **Abstract**

Cancer continues to be one of the main public health challenges, driving the search for new compounds with therapeutic potential. Medicinal plants represent a valuable, promising source of bioactive metabolites, and the Brine Shrimp Lethality Test has been widely used as a preliminary tool to assess the toxicity of natural extracts, providing clues to their possible anticancer activity. In this study, the cytotoxicity of the extracts of *Annona stenophylla* (Engl. & Diels), *Strophanthus petersianus* (Klotzsch), and *Synadenium glaucescens* (Pax) was investigated using the BSLT as a first step in screening for potential anticancer compounds. The plant materials were harvested in Tanzania and air-dried in the shade and ground. The extracts were prepared by total sequential solvent extraction using cold maceration, starting with ethyl acetate followed by methanol. A total of 24 ethyl acetate and methanolic extracts were obtained from the leaves, stem bark, stem wood, root wood and root bark of the three plants studied. The toxicity of the extracts was assessed by exposing *Artemia salina* nauplii to different concentrations of the extracts, with mortality recorded after 24 hours. The LC_50_ was determined to evaluate the toxicity of each extract. All the extracts from the three plants exhibited different degrees of toxicity, with *A. stenophylla* demonstrating the lowest LC_50_ values, indicating the highest toxicity. The methanolic extract of *A. stenophylla*’s root wood exhibited the highest toxicity, producing a mortality rate of 99.44%, corresponding to an LC_50_ < 20 μg/mL. The observed toxicity suggests the presence of bioactive compounds with potential anticancer activities. The results support the potential of *A. stenophylla*, *S. petersianus* and *S. glaucescens* as sources of bioactive compounds with possible anticancer activity. Further studies, including phytochemical analysis and *in vitro* anticancer assays, are recommended to identify and characterize the active constituents responsible for the observed cytotoxic effects.

**Keywords**: Brine Shrimp Lethality Test, cytotoxicity, *Annona stenophylla*, *Strophanthus petersianus* and *Synadenium glaucescens*, medicinal plants, anticancer screening.

# **S1 Appendix 1: Detailed description of the method used to prepare artificial sea salt from seawater collected from the Indian Ocean**

Artificial sea salt was prepared by the Laboratory of the Department of Physics and Chemistry at Sokoine University of Agriculture. Approximately 25 liters of seawater were collected from the Indian Ocean in Dar es Salaam. The water was filtered to remove particulates such as sand, debris, and small organisms. It was then boiled in a large pot over medium-high heat to allow gradual evaporation. As the water evaporated, salt crystals began to form. Once most of the water had evaporated, the heat was turned off, and the crystals were collected from the bottom and sides of the pot. The harvested salt was spread on glass trays and left to air dry. Finally, the dried sea salt was packed into a clean jar for storage.

# **S1 Tables 1-26: Raw data on the survival of Artemia salina larvae after 24 hours of exposure to different concentrations of extracts and control treatments**

**Table 1. Root bark of *A. stenophylla* EtOAc**

| **Conc. μg/mL** | **Rep 1** | **Rep 2** | **Rep 3** | **Average** | **% mortality** |
| --- | --- | --- | --- | --- | --- |
| 20 | 5 | 3 | 1 | 3.0 | 70.0 |
| 40 | 0 | 1 | 0 | 0.3 | 96.7 |
| 80 | 0 | 0 | 0 | 0.0 | 100.0 |
| 180 | 0 | 0 | 0 | 0.0 | 100.0 |
| 240 | 0 | 0 | 0 | 0.0 | 100.0 |
| 360 | 0 | 0 | 0 | 0.0 | 100.0 |

**Table 2. Root bark of *A. stenophylla* MeOH extract**

| **Conc. μg/mL** | **Rep 1** | **Rep 2** | **Rep 3** | **Average** | **% mortality** |
| --- | --- | --- | --- | --- | --- |
| 20 | 8 | 8 | 9 | 8.3 | 16.7 |
| 40 | 8 | 6 | 8 | 7.3 | 26.7 |
| 80 | 7 | 7 | 7 | 7.0 | 30.0 |
| 180 | 7 | 5 | 6 | 6.0 | 40.0 |
| 240 | 1 | 2 | 2 | 1.7 | 83.3 |
| 360 | 1 | 1 | 1 | 1.0 | 90.0 |

**Table 3. Root wood *A. stenophylla* EtOAc extract**

| **Conc. μg/mL** | **Rep 1** | **Rep 2** | **Rep 3** | **Average** | **% mortality** |
| --- | --- | --- | --- | --- | --- |
| 20 | 2 | 1 | 1 | 1.3 | 86.7 |
| 40 | 0 | 0 | 1 | 0.3 | 96.7 |
| 80 | 0 | 0 | 0 | 0.0 | 100.0 |
| 180 | 0 | 0 | 0 | 0.0 | 100.0 |
| 240 | 0 | 0 | 0 | 0.0 | 100.0 |
| 360 | 0 | 0 | 0 | 0.0 | 100.0 |

**Table 4. Root wood *A. stenophylla* MeOH extract**

| **Conc. μg/mL** | **Rep 1** | **Rep 2** | **Rep 3** | **Average** | **% mortality** |
| --- | --- | --- | --- | --- | --- |
| 20 | 0 | 0 | 1 | 0.3 | 96.7 |
| 40 | 0 | 0 | 0 | 0.0 | 100.0 |
| 80 | 0 | 0 | 0 | 0.0 | 100.0 |
| 180 | 0 | 0 | 0 | 0.0 | 100.0 |
| 240 | 0 | 0 | 0 | 0.0 | 100.0 |
| 360 | 0 | 0 | 0 | 0.0 | 100.0 |

**Table 5. Stems *A. stenophylla* EtOAc extract**

| **Conc. μg/mL** | **Rep 1** | **Rep 2** | **Rep 3** | **Average** | **% mortality** |
| --- | --- | --- | --- | --- | --- |
| 20 | 1 | 3 | 2 | 2.0 | 80.0 |
| 40 | 0 | 1 | 1 | 0.7 | 93.3 |
| 80 | 0 | 0 | 0 | 0.0 | 100.0 |
| 180 | 0 | 0 | 0 | 0.0 | 100.0 |
| 240 | 0 | 0 | 0 | 0.0 | 100.0 |
| 360 | 0 | 0 | 0 | 0.0 | 100.0 |

**Table 6. Stems *A. stenophylla* MeOH extract**

| **Conc. μg/mL** | **Rep 1** | **Rep 2** | **Rep 3** | **Average** | **% mortality** |
| --- | --- | --- | --- | --- | --- |
| 20 | 3 | 3 | 1 | 2.3 | 76.7 |
| 40 | 0 | 0 | 1 | 0.3 | 96.7 |
| 80 | 0 | 0 | 0 | 0.0 | 100.0 |
| 180 | 0 | 0 | 0 | 0.0 | 100.0 |
| 240 | 0 | 0 | 0 | 0.0 | 100.0 |
| 360 | 0 | 0 | 0 | 0.0 | 100.0 |

**Table 7. Leaves *A. stenophylla* EtOAc extract**

| **Conc. μg/mL** | **Rep 1** | **Rep 2** | **Rep 3** | **Average** | **% mortality** |
| --- | --- | --- | --- | --- | --- |
| 20 | 4 | 5 | 6 | 5.0 | 50.0 |
| 40 | 5 | 3 | 4 | 4.0 | 60.0 |
| 80 | 1 | 0 | 2 | 1.0 | 90.0 |
| 180 | 0 | 2 | 0 | 0.7 | 93.3 |
| 240 | 0 | 0 | 0 | 0.0 | 100.0 |
| 360 | 0 | 0 | 0 | 0.0 | 100.0 |

**Table 8. Leaves *A. stenophylla* MeOH extract**

| **Conc. μg/mL** | **Rep 1** | **Rep 2** | **Rep 3** | **Average** | **% mortality** |
| --- | --- | --- | --- | --- | --- |
| 20 | 5 | 6 | 7 | 6.0 | 40.0 |
| 40 | 6 | 5 | 6 | 5.7 | 43.3 |
| 80 | 7 | 3 | 5 | 5.0 | 50.0 |
| 180 | 2 | 5 | 5 | 4.0 | 60.0 |
| 240 | 4 | 4 | 4 | 4.0 | 60.0 |
| 360 | 5 | 4 | 3 | 4.0 | 60.0 |

**Table 9. Roots of *S. petersianus* EtOAc extract**

| **Conc. μg/mL** | **Rep 1** | **Rep 2** | **Rep 3** | **Average** | **% mortality** |
| --- | --- | --- | --- | --- | --- |
| 20 | 8 | 9 | 9 | 8.7 | 13.3 |
| 40 | 6 | 9 | 9 | 8.0 | 20.0 |
| 80 | 6 | 7 | 7 | 6.7 | 33.3 |
| 180 | 6 | 7 | 7 | 6.7 | 33.3 |
| 240 | 6 | 7 | 7 | 6.7 | 33.3 |
| 360 | 4 | 6 | 5 | 5.0 | 50.0 |

**Table 10. Roots of *S. petersianus* MeOH extract**

| **Conc. μg/mL** | **Rep 1** | **Rep 2** | **Rep 3** | **Average** | **% mortality** |
| --- | --- | --- | --- | --- | --- |
| 20 | 10 | 10 | 10 | 10.0 | 0.0 |
| 40 | 10 | 10 | 9 | 9.7 | 3.3 |
| 80 | 9 | 10 | 9 | 9.3 | 6.7 |
| 180 | 8 | 9 | 8 | 8.3 | 16.7 |
| 240 | 7 | 8 | 6 | 7.0 | 30.0 |
| 360 | 5 | 6 | 4 | 5.0 | 50.0 |

**Table 11. Stems of *S. petersianus* EtOAc extract**

| **Conc. μg/mL** | **Rep 1** | **Rep 2** | **Rep 3** | **Average** | **% mortality** |
| --- | --- | --- | --- | --- | --- |
| 20 | 10 | 10 | 9 | 9.7 | 3.3 |
| 40 | 9 | 10 | 9 | 9.3 | 6.7 |
| 80 | 10 | 9 | 9 | 9.3 | 6.7 |
| 180 | 9 | 8 | 8 | 8.3 | 16.7 |
| 240 | 8 | 7 | 8 | 7.7 | 23.3 |
| 360 | 5 | 6 | 7 | 6.0 | 40.0 |

**Table 12. Stems of *S. petersianus* MeOH extract**

| **Conc. μg/mL** | **Rep 1** | **Rep 2** | **Rep 3** | **Average** | **% mortality** |
| --- | --- | --- | --- | --- | --- |
| 20 | 8 | 9 | 9 | 8.7 | 13.3 |
| 40 | 8 | 7 | 9 | 8.0 | 20.0 |
| 80 | 7 | 7 | 6 | 6.7 | 33.3 |
| 180 | 6 | 6 | 6 | 6.0 | 40.0 |
| 240 | 6 | 5 | 5 | 5.3 | 46.7 |
| 360 | 5 | 4 | 6 | 5.0 | 50.0 |

**Table 13. Leaves of *S. petersianus* EtOAc extract**

| **Conc. μg/mL** | **Rep 1** | **Rep 2** | **Rep 3** | **Average** | **% mortality** |
| --- | --- | --- | --- | --- | --- |
| 20 | 10 | 10 | 10 | 10 | 0.0 |
| 40 | 10 | 10 | 10 | 10 | 0.0 |
| 80 | 10 | 10 | 10 | 10 | 0.0 |
| 180 | 10 | 10 | 10 | 10 | 0.0 |
| 240 | 9 | 9 | 10 | 9.3 | 6.7 |
| 360 | 8 | 9 | 9 | 8.7 | 13.3 |

**Table 14. Leaves of *S. petersianus* MeOH extract**

| **Conc. μg/mL** | **Rep 1** | **Rep 2** | **Rep 3** | **Average** | **% mortality** |
| --- | --- | --- | --- | --- | --- |
| 20 | 10 | 10 | 10 | 10.0 | 0.0 |
| 40 | 10 | 9 | 10 | 9.7 | 3.3 |
| 80 | 9 | 8 | 8 | 8.3 | 16.7 |
| 180 | 7 | 7 | 6 | 6.7 | 33.3 |
| 240 | 5 | 6 | 7 | 6.0 | 40.0 |
| 360 | 4 | 5 | 5 | 4.7 | 53.3 |

**Table 15. Root bark of *S. glaucescens* EtOAc extract**

| **Conc. μg/mL** | **Rep 1** | **Rep 2** | **Rep 3** | **Average** | **% mortality** |
| --- | --- | --- | --- | --- | --- |
| 20 | 1 | 4 | 4 | 3.0 | 70.0 |
| 40 | 2 | 2 | 1 | 1.7 | 83.3 |
| 80 | 2 | 3 | 1 | 2.0 | 80.0 |
| 180 | 2 | 2 | 1 | 1.7 | 83.3 |
| 240 | 1 | 2 | 2 | 1.7 | 83.3 |
| 360 | 0 | 0 | 1 | 0.3 | 96.7 |

**Table 16. Root bark of *S. glaucescens* MeOH extract**

| **Conc. μg/mL** | **Rep 1** | **Rep 2** | **Rep 3** | **Average** | **% mortality** |
| --- | --- | --- | --- | --- | --- |
| 20 | 6 | 9 | 8 | 7.7 | 23.3 |
| 40 | 6 | 6 | 4 | 5.3 | 46.7 |
| 80 | 4 | 3 | 4 | 3.7 | 63.3 |
| 180 | 3 | 3 | 3 | 3.0 | 70.0 |
| 240 | 4 | 3 | 3 | 3.3 | 66.7 |
| 360 | 4 | 3 | 3 | 3.3 | 66.7 |

**Table 17. Root wood of *S. glaucescens* EtOAc extract**

| **Conc. μg/mL** | **Rep 1** | **Rep 2** | **Rep 3** | **Average** | **% mortality** |
| --- | --- | --- | --- | --- | --- |
| 20 | 4 | 5 | 4 | 4.3 | 56.7 |
| 40 | 3 | 2 | 3 | 2.7 | 73.3 |
| 80 | 1 | 3 | 1 | 1.7 | 83.3 |
| 180 | 0 | 1 | 0 | 0.3 | 96.7 |
| 240 | 0 | 0 | 0 | 0.0 | 100.0 |
| 360 | 0 | 0 | 0 | 0.0 | 100.0 |

**Table 18. Root wood of *S. glaucescens* MeOH extract**

| **Conc. μg/mL** | **Rep 1** | **Rep 2** | **Rep 3** | **Average** | **% mortality** |
| --- | --- | --- | --- | --- | --- |
| 20 | 2 | 2 | 2 | 2.0 | 80.0 |
| 40 | 2 | 2 | 1 | 1.7 | 83.3 |
| 80 | 2 | 1 | 1 | 1.3 | 86.7 |
| 180 | 0 | 1 | 1 | 0.7 | 93.3 |
| 240 | 1 | 0 | 0 | 0.3 | 96.7 |
| 360 | 0 | 0 | 0 | 0.0 | 100.0 |

**Table 19. Stem bark of *S. glaucescens* EtOAc extract**

| **Conc. μg/mL** | **Rep 1** | **Rep 2** | **Rep 3** | **Average** | **% mortality** |
| --- | --- | --- | --- | --- | --- |
| 20 | 3 | 5 | 7 | 5.0 | 50.0 |
| 40 | 3 | 3 | 4 | 3.3 | 66.7 |
| 80 | 3 | 3 | 3 | 3.0 | 70.0 |
| 180 | 2 | 2 | 2 | 2.0 | 80.0 |
| 240 | 2 | 1 | 2 | 1.7 | 83.3 |
| 360 | 0 | 0 | 0 | 0.0 | 100.0 |

**Table 20. Stem bark of *S. glaucescens* MeOH extract**

| **Conc. μg/mL** | **Rep 1** | **Rep 2** | **Rep 3** | **Average** | **% mortality** |
| --- | --- | --- | --- | --- | --- |
| 20 | 10 | 10 | 10 | 10.0 | 0.0 |
| 40 | 10 | 9 | 10 | 9.7 | 3.3 |
| 80 | 9 | 10 | 10 | 9.7 | 3.3 |
| 180 | 9 | 9 | 9 | 9.0 | 10.0 |
| 240 | 9 | 7 | 9 | 8.3 | 16.7 |
| 360 | 8 | 7 | 8 | 7.7 | 23.3 |

**Table 21. Stem wood of *S. glaucescens* EtOAc extract**

| **Conc. μg/mL** | **Rep 1** | **Rep 2** | **Rep 3** | **Average** | **% mortality** |
| --- | --- | --- | --- | --- | --- |
| 20 | 10 | 10 | 10 | 10.0 | 0.0 |
| 40 | 10 | 9 | 10 | 9.7 | 3.3 |
| 80 | 9 | 10 | 10 | 9.7 | 3.3 |
| 180 | 9 | 9 | 9 | 9.0 | 10.0 |
| 240 | 9 | 7 | 9 | 8.3 | 16.7 |
| 360 | 8 | 7 | 8 | 7.7 | 23.3 |

**Table 22. Stem wood of *S. glaucescens* MeOH extract**

| **Conc. μg/mL** | **Rep 1** | **Rep 2** | **Rep 3** | **Average** | **% mortality** |
| --- | --- | --- | --- | --- | --- |
| 20 | 8 | 8 | 7 | 7.7 | 23.3 |
| 40 | 8 | 8 | 7 | 7.7 | 23.3 |
| 80 | 7 | 7 | 6 | 6.7 | 33.3 |
| 180 | 4 | 3 | 4 | 3.7 | 63.3 |
| 240 | 4 | 3 | 4 | 3.7 | 63.3 |
| 360 | 3 | 3 | 4 | 3.3 | 66.7 |

**Table 23. Leaves of *S. glaucescens* EtOAc extract**

| **Conc. μg/mL** | **Rep 1** | **Rep 2** | **Rep 3** | **Average** | **% mortality** |
| --- | --- | --- | --- | --- | --- |
| 20 | 9 | 8 | 8 | 8.3 | 16.7 |
| 40 | 8 | 7 | 8 | 7.7 | 23.3 |
| 80 | 7 | 7 | 8 | 7.3 | 26.7 |
| 180 | 6 | 5 | 5 | 5.3 | 46.7 |
| 240 | 5 | 5 | 6 | 5.3 | 46.7 |
| 360 | 4 | 3 | 3 | 3.3 | 66.7 |

**Table 24. Leaves of *S. glaucescens* MeOH extract**

| **Conc. μg/mL** | **Rep 1** | **Rep 2** | **Rep 3** | **Average** | **% mortality** |
| --- | --- | --- | --- | --- | --- |
| 20 | 8 | 7 | 8 | 7.7 | 23.3 |
| 40 | 8 | 7 | 8 | 7.7 | 23.3 |
| 80 | 7 | 8 | 7 | 7.3 | 26.7 |
| 180 | 6 | 6 | 6 | 6.0 | 40.0 |
| 240 | 5 | 5 | 6 | 5.3 | 46.7 |
| 360 | 5 | 4 | 5 | 4.7 | 53.3 |

**Table 25. Positive control - Leaves of *Catharanthus roseus* MeOH extract**

| **Conc. μg/mL** | **Rep 1** | **Rep 2** | **Rep 3** | **Average** | **% mortality** |
| --- | --- | --- | --- | --- | --- |
| 20 | 10 | 10 | 10 | 10 | 0.0 |
| 40 | 10 | 10 | 10 | 10 | 0.0 |
| 80 | 10 | 9 | 9 | 9.3 | 6.7 |
| 180 | 9 | 8 | 9 | 8.7 | 13.3 |
| 240 | 6 | 7 | 6 | 6.3 | 36.7 |
| 360 | 4 | 5 | 6 | 5 | 50.0 |

**Table 26. Negative control (1% DMSO)**

| **Conc. μg/mL** | **Rep 1** | **Rep 2** | **Rep 3** | **Average** | **% mortality** |
| --- | --- | --- | --- | --- | --- |
| 20 | 10 | 10 | 10 | 10 | 0 |
| 40 | 10 | 10 | 10 | 10 | 0 |
| 80 | 10 | 10 | 10 | 10 | 0 |
| 180 | 10 | 10 | 10 | 10 | 0 |
| 240 | 10 | 10 | 10 | 10 | 0 |
| 360 | 9 | 10 | 10 | 9.7 | 3.3 |
