# Supplementary material for: Screening anticancer activity by Brine shrimp lethality test of extracts of Annona stenophylla (Engl. & Diels), Strophanthus petersianus (Klotzsch) and Synadenium glaucescens (Pax)
Source: PLoS One. 2026 Jan 2;21(1):e0336636. doi: 10.1371/journal.pone.0336636 (PMC12758728; doi:10.1371/journal.pone.0336636)
Supplement: Attachment — Submitted filename: Response to Reviewers.docx [file pone.0336636.s003.docx]

# **Journal requirements**

When submitting your revision, we need you to address these additional requirements.

1. Please ensure that your manuscript meets PLOS ONE's style requirements, including those for file naming. The PLOS ONE style templates can be found at

**Response**: We thank the editorial team for the guidance. We have revised the manuscript and ensured that it conforms to the PLOS ONE style and formatting requirements. All files have been renamed according to the PLOS ONE file-naming conventions.

2. In your Methods section, please provide additional information regarding the permits you obtained for the work. Please ensure you have included the full name of the authority that approved the field site access and, if no permits were required, a brief statement explaining why.

**Response**: We thank the editorial team for this comment. The Methods section has been updated to include detailed information on the field permit. Fieldwork was conducted with permission from the Directorate of Postgraduate Studies, Research, Technology Transfer and Consultancy (DPRTC) of Sokoine University of Agriculture, under reference SUA/DPRTC/PYT/D/2022/0001/08.

No additional permits were needed beyond the one issued by the DPRTC of Sokoine University of Agriculture.

3.  We note that the grant information you provided in the ‘Funding Information’ and ‘Financial Disclosure’ sections does not match.

When you resubmit, please ensure that you provide the correct grant numbers for the awards you received for your study in the ‘Funding Information’ section.

**Response**: We thank the editorial team for noting this discrepancy. We have corrected the information to ensure consistency between the ‘Funding Information’ and ‘Financial Disclosure’ sections.

4. Thank you for stating the following financial disclosure:

[This research was supported by the Partnership for Skills in Applied Sciences, Engineering and Technology - Regional Scholarship and Innovation Fund (PASET-RSIF) scholarship 2022 grant no. P165581].

Please state what role the funders took in the study.  If the funders had no role, please state: ""The funders had no role in study design, data collection and analysis, decision to publish, or preparation of the manuscript.""

If this statement is not correct, you must amend it as needed.

Please include this amended Role of Funder statement in your cover letter; we will change the online submission form on your behalf.

**Response**: Thank you for the feedback. We have amended the statement to accurately reflect the revised information:

*This study was funded by the Regional Scholarship and Innovation Fund (RSIF) of the Partnership for Skills in Applied Sciences, Engineering and Technology (PASET) (Scholarship 2022 grant N^ο^ P165581) grant to SACIDS Africa Centre of Excellence for Infectious Diseases of Human and Animals in Southern and Eastern Africa (SACIDS-ACE) at the Sokoine University of Agriculture (SUA). Roberto Luis Nhamussua is a recipient of an RSIF-PASET doctoral scholarship at SUA.*

5. We note that your Data Availability Statement is currently as follows: [All relevant data are within the manuscript and its Supporting Information files.]

Please confirm at this time whether or not your submission contains all raw data required to replicate the results of your study. Authors must share the “minimal data set” for their submission. PLOS defines the minimal data set to consist of the data required to replicate all study findings reported in the article, as well as related metadata and methods (<https://journals.plos.org/plosone/s/data-availability#loc-minimal-data-set-definition>).

For example, authors should submit the following data:

- The values behind the means, standard deviations and other measures reported;

- The values used to build graphs;

- The points extracted from images for analysis.

Authors do not need to submit their entire data set if only a portion of the data was used in the reported study.

If your submission does not contain these data, please either upload them as Supporting Information files or deposit them to a stable, public repository and provide us with the relevant URLs, DOIs, or accession numbers. For a list of recommended repositories, please see <https://journals.plos.org/plosone/s/recommended-repositories>.

If there are ethical or legal restrictions on sharing a de-identified data set, please explain them in detail (e.g., data contain potentially sensitive information, data are owned by a third-party organization, etc.) and who has imposed them (e.g., an ethics committee). Please also provide contact information for a data access committee, ethics committee, or other institutional body to which data requests may be sent. If data are owned by a third party, please indicate how others may request data access.

6. Please include your full ethics statement in the ‘Methods’ section of your manuscript file. In your statement, please include the full name of the IRB or ethics committee who approved or waived your study, as well as whether or not you obtained informed written or verbal consent. If consent was waived for your study, please include this information in your statement as well.

**Response**: We thank the reviewer for the guidance. We have now included the full ethics statement in the 'Methods' section of the manuscript. The addition made in the methods section of the manuscript is on lines 165-171 and reads as follows:

## ***Ethics statement***

*“This study did not involve human participants or vertebrate animals and therefore did not require ethical approval. Field collection of plant material was conducted with permission from the Directorate of Postgraduate Studies, Research, Technology Transfer and Consultancy (DPRTC) of Sokoine University of Agriculture under reference number SUA/DPRTC/PYT/D/2022/0001/08. The study complied with all institutional, national, and international guidelines for the collection and use the plant materials in research”.*

## **Reviewer comment and author's response**

# **Introduction**

Line 52-53. I assume that the authors are saying that the names of different cancers come from the different organs and tissues in which uncontrolled cell growth occurs, but with the words “it is” and “they are found” this makes the sentence awkward to read, please reword.

**Response**: Thank you for pointing this out. We have revised the sentence for clarity. The revised sentence now on lines 50-51 reads as follows:

“*Different types of cancer are typically named according to the type of tissue or organ in which they originate*”

Line 54, please add a comma behind the word ‘unknown’ to assist with grammar and sentence structure “are still unknown, nevertheless, factors such as…”
**Response**: Thank you for your careful reading and helpful suggestion. We have revised the sentence on lines 52-54 to read as follows:

“*The causes of most cancers are still unknown, nevertheless, factors such as lifestyle habits like smoking, excessive body weight gained, hormonal contributions, as well as non-modifiable factors like genetic aberration, were among the major causes*”

Line 60-62, could the authors include a short description of some of the adverse effects that current cancer treatments have? Or could the impact/level of “adverse” be described here? As a reader, I like that the sentence is citing other works that describe these effects, however, to read a small blip about what those are and why they are important to this current study piques my interest more than a simple citation.

**Response**: Thank you for this insightful suggestion. We have added a brief description of common adverse effects associated with current cancer treatments to provide additional context. The revised passage on lines 58-62 now reads as follows:

“*Therefore, new strategies or compounds need to be discovered to provide effective treatment with fewer adverse effects because most cancer treatments, such as chemotherapy and radiotherapy can cause fatigue, nausea, vomiting, hair loss, dizziness, lack of appetite and others, which in most cases, lead to the patient abandoning the treatment*”

Line 64: What are the authors referring to here when they say “natural products”?

**Response:** Thank you for pointing this out. We mean by natural products bioactive compounds derived from plants, microorganisms or marine organisms. To improve clarity, we have updated the sentence on lines 62-63, now reads as follows:

*“Medicinal plants have been used for years and widely explored as one of the natural sources of active compounds for treating cancer and other diseases”*

Line 76, please remove the word “which” from this sentence to improve grammar and sentence structure.

**Response:** Thank you for your suggestion. We have revised the sentence by removing the word which to improve grammar and sentence flow. The updated sentence on lines 74-75 now reads as follows:

*“A. stenophylla belongs to the Annonaceae family and is known for its bioactive metabolites such as acetogenins, flavonoids and alkaloids with cytotoxicity and anticancer potential”*

Line 77-79, please reword this sentence. The comma usage as well as general word choice and syntax make this very difficult to read and understand.

**Response:** Thank you for highlighting this issue. We have revised it to improve readability and clarity. The new sentence on lines 76-77 now reads as follows:

*“S. petersianus is a member of the Apocynaceae family and belongs to a group of plants traditionally used in African medicine to treat many diseases, including cancer.”*

Line 84, I believe the word here should be “including” rather than “inclining”.

**Response**: Thank you for catching this error. We have made the correction accordingly and the revised sentence on lines 81-82 now reads as follows:

*“Due to its bioactive compounds, S. glaucescens, from the Euphorbiaceae family, has been used in traditional medicine for various diseases, including cancer.”*

Line 82-85. The end of this sentence needs a re-wording as singular and plural versions of “agents” are being described. If euphol and lupeol and B-sitosterol are anticancer agents then they are “candidates” for cancer treatment, not “the candidate”. If only one is being mentioned as a prime candidate, then the sentence needs to be restructured.

**Response**: Thank you for the helpful observation. We have revised the sentence to correctly reflect the plural form and improve clarity. The updated sentence on lines 82-85 now reads as follows:

*”For instance, Euphol and Lupeol are the terpenes isolated from S. grantii and glaucescens and β-sitosterol, a steroid isolated from diverse species including S. glaucescens, are anticancer agents and therefore candidates for cancer treatment.”*

In addition, we thought it necessary to provide some details about Euphol, Lupeol and β-sitosterol to clarify why they could be considered candidates for cancer treatment. The updated brief on lines 85-89 reads as follows:

*“Further, Euphol revealed in vitro cytotoxicity against B16F10 melanoma cell lines and in vivo assay showed significant reduction in tumour volume in melanoma-bearing mice, while Lupeol demonstrated anti-neoplastic effects against A549, a human non-small cell lung cancer cell line, and β-sitosterol exhibited cytotoxic activity against MCF-7 cancer cell lines”.*

Line 86, Sentence rewording suggestion to improve clarity: “In Tanzania, *A. stenophylla*’s fruits are highly sought after by herdsmen and children as these fruits produce non-alcoholic juice”. I had to read this twice to understand what was being stated.

**Response:** Thank you for pointing this out. We have revised it as follows (lines 94-96):

*“In Tanzania, A. stenophylla can be found in Western, Rukwa, Tabora and Iringa Regions, and its fruits are particularly appreciated by herdsmen and children, who consume them for their naturally sweet, non-alcoholic juice”*

Lines 86-96. I really like how the authors describe each of these plants’ origins, their common/medicinal uses, where they are native to and provide each of the citations for this information, I only wish that it was consistent for each of the species. I would like the common name for all of them, not only just the sand forest poison rope, and I believe that just for structure of this paragraph, if the authors keep a consistent format for how they introduce each of these species and the corresponding information, it would flow better and be more collectively informative.

**Response**: We thank the reviewer for this helpful and encouraging comment. We agree that the consistency in the presentation of the plant species would improve the clarity and the flow of the paragraph. We have revised the format to ensure consistency for each species. The updated format on lines 90-108 reads as follows:

*“A. stenophylla is commonly known as the dwarf custard apple in English, and is referred to as Mtopetope in Swahili, particularly in Tanzania. The species has been recorded in Tanzania, Zambia, Zimbabwe, Angola, Botswana, Mozambique, the Republic Democratic of Congo and Namibia in woodland and sandy grassy slopes at the edge of wetlands. In Tanzania, A. stenophylla can be found in Western, Rukwa, Tabora and Iringa Regions [26], and its fruits are particularly appreciated by herdsmen and children, who consume them for their naturally sweet, non-alcoholic juice [11]. The plant is commonly used in folk therapy as a snake repellent, for body swelling, managing diabetes, constipation, stomach pains, chest pain, blood purification, menorrhagia, dysmenorrhea, gonorrhoea, syphilis, antiemetic and muscle sprains.*

*The species S. petersianus, commonly known as the sand forest poison rope, has been used as a poison for arrows and by the Zulus as an amulet against evil. It is native to countries from southern Kenya to South Africa.*

*S. glaucescens is commonly known as the milk bush plant in English and Mvunjakongwa in Swahili. The plant is endemic in the East African Region and occurs in Tanzania, Kenya, the Democratic Republic of Congo and Burundi. In Tanzania, it is distributed across diverse regions and is traditionally used for treating wounds, skin therapy, toothache, cough, tuberculosis, sexually transmitted infections, Human Immunodeficiency Virus (HIV), gastrointestinal worms and ringworms, excessive menstruation and asthma therapy”*

Line 97, please correct from “…the toxicity of the extract…” to “…the toxicity of an extract…”
**Response**: We thank the reviewer for this careful observation. We have revised the phrase on line 97 as suggested, changing the toxicity of the extract to the toxicity of an extract to improve the clarity and accuracy. The revised version on line 109 reads as follows:

*“The BSLT method is the first step for testing the toxicity of an extract or compound”*

Lines 97 to 100, these two sentences could be combined with a semi-colon as they relate closely to one another and are also both independent clauses.

**Response**: We appreciate the reviewer’s suggestion concerning sentence structure in lines 97-100. We have revised these sentences by combining them with a semi-colon, as recommended to enhance the flow and cohesion. The change made in the manuscript, on lines 109-110, reads as follows:

*“The BSLT method is the first step for testing the toxicity of an extract or compound; it is also used to determine the bioactivity of a compound from a natural product”.*

Lines 107-111. This sentence is far too long, and the incorrect comma usage makes it difficult to understand. Please reword.

**Response**: We sincerely appreciate your time regarding clarity and sentence structure. We have revised the sentence to improve readability and breaking it into short sentences and correcting comma usage. The change made in the manuscript on lines 119-122 reads as follows:

*“Previous studies of A. stenophylla have assessed the antioxidant activity of its extracts. On another hand, researchers have investigated whether the root extract can inhibit α-glucosidase and α-amylase enzymes in the presence of carbohydrate substrates, suggesting a possible mechanism for its antidiabetic activity. Additional studies include acute and subacute toxicity tests of its roots using rats, as well as screening its roots and leaves using the BSLT method.”*

Line 113. A study was conducted on what that assessed toxicity of each extract mentioned? Using a citation number to replace words within a sentence is not appropriate, please keep the citation, but also describe for the reader what you are referring to and the point which you are trying to make.

**Response:** We appreciate the reviewer’s helpful suggestion. We have revised the sentence to specify what the study assessed, rather than relying solely on a citation number. The revised sentence includes a brief description of the study focus to ensure the context and relevance are clear to the reader. The updated sentence on lines 124-127 now reads as follows:

*“Among the various studies conducted on S. glaucescens, Mabiki (2013) assessed the toxicity of extracts from the root bark, root wood, stem bark, stem wood and leaves using the BSLT method. The findings suggested that these extracts possess potential anticancer properties”.*

# **Materials and methods**

Lines 125 and 127, as the genus and species names have already been fully introduced within the introduction section, each plant species here can be referred to in the abbreviated manner “*A. stenophylla, S. petersianus*, and *S. glaucescens*”.

**Response**: We thank the reviewer for this valuable comment. We have revised the text to use abbreviated forms of the species names. The change was made in the manuscript on lines 136-138 to replace the full name with the abbreviated forms as follows:

“*A. stenophylla,* *S. petersianus* and *S. glaucescens”.*

Line 136. Please add a comma after the word “compounds, …”

**Response:** We thank the reviewer for the careful reading. As suggested, we have added a comma after the word “compounds” to improve clarity. The addition made in the manuscript is on line 154 and reads as follows.

*“To avoid the decomposition of temperature light-sensitive compounds, all collected plant materials were air-dried under the shade…”*

Line 138. As I, and I assume others, am unfamiliar with the method of harvesting and drying plants, could the authors describe this process in a little more detail here? How long were the plants dried in the shade, what length of time did the weight/mass need to remain consistent to be considered “dry”, did the bark and wood take longer than the roots, stems, and leaves? How did the “harvesting” actually occur, were tools used or was it a delicate hand-picking process? Was the plant matter collected in a different manner than the bark and wood?

**Response**: We truly appreciate the reviewer’s thoughtful comment and the opportunity to clarify our methodology. We have expanded the description in the manuscript to include more detail on the harvesting and drying process. The updates made in the manuscript on lines 144-162 now read as follows:

*“The plants under study were collected using sustainable methods, which involved harvesting only the specific parts required for the research. This approach aimed to minimise waste, prevent overharvesting, and ensure the long-term preservation of plant populations. All species were accurately identified by qualified botanists, and representative samples were conserved for future research. Plant materials were manually harvested using appropriate tools such as pruning shears and small knives, depending on the plant part. Leaves were handpicked to avoid damaging delicate structures; stems and their bark were carefully cut using pruning shears. Similarly, roots were excavated with hoes and subsequently sectioned along with their bark using machetes. Fruits were not available at the time of collection. All materials were placed in breathable paper bags to avoid moisture retention during transport”*.

*“To avoid the decomposition of temperature and light-sensitive compounds, all collected plant materials were air-dried under the shade (approximately 25 °C) in the Department of Chemistry and Physics laboratory at the Sokoine University of Agriculture. The plant materials were dried until they achieved a constant weight, determined over three consecutive days of weighing. The leaves of A. stenophylla and S. petersianus required 12 days to reach this point, whereas the leaves of S. glaucescens, due to their higher moisture content, required 23 days to reach a constant weight. The bark and wood of A. stenophylla and S. glaucescens were dried separately, and they took approximately 15 days to dry completely. For S. petersianus, the root and stem were not separated from their bark and required 15 days to dry”.*

Line 140. I believe “Silver Crest reference” relates to the electric mill device being used to ground the dried materials, but I am not sure if that is part of the name of the device or what purpose these words have within the sentence.

**Response:** Thank you for your comment regarding the phrase “Silver Crest reference” on line 165. We confirm that refers to the brand name of the electric mill used to grind the dried materials. The intention was to provide a clear identification of the equipment employed in our study to enhance reproducibility. We have revised to clarity as follows (lines 162-164):

*“The dried materials were ground using an electric mill (Silver Crest brand) to ensure uniform particle size, then weighed and packaged in polyethylene zipper bags”.*

Line 143-146. I believe this description of plant species, parts, and their specific masses would be better described visually with a small table rather than in sentence format. It would significantly reduce the length of the first sentence of the extraction paragraph also. Suggestion: “Harvested plant parts (Table 1) were subjected to total and sequential cold maceration starting with ethyl acetate, followed by methanol as solvents”.

**Response**: Thank you for your valuable suggestion regarding lines 143-146 in the original manuscript. We have reformatted this information into Table 2, which allows for a more concise reader reader-friendly presentation. The change was made in the manuscript on lines 173-175 and reads as follows:

*“Each harvested plant part (Table 2) was subjected to total and sequential solvent extraction using cold maceration, starting with ethyl acetate (EtOAc) to extract low to medium polarity compounds, followed by methanol (MeOH) to obtain more polar constituents”*

***Table 2****: Harvested plant parts and corresponding masses used for extraction*

| *species /parts* | *Root bark* | *Root wood* | *Stems bark* | *Stems wood* | *Leaves* |
| --- | --- | --- | --- | --- | --- |
| *A. stenophylla* | *1500 g* | *2400 g* | *1500 g* | | *1900 g* |
| *S. petersianus* | *1400 g* | | *2000 g* | | *1700 g* |
| *S. glaucescens* | *2000 g* | *1600 g* | *1000 g* | *1600 g* | *1000 g* |

Line 147. Was 300 g or 400 g used per 2 L of solvent? Did it depend on which plant part was being macerated? Was the solvent a 1:1 ratio of ethyl acetate and methanol? Why 2 liters of solvent per 300-400g of solute, was this an established in-house protocol for extraction?

**Response**: We thank you for your insightful questions. We appreciate the opportunity to clarify the methodology. A total of 300-400 g of plant material was used per 2 L of solvent, with the exact quantity depending on the availability and density of each plant part. This was done to maintain a consistent solvent-to-solute ratio that ensures adequate solvent penetration and efficient extraction. The solvents were not used as a mixture; rather, cold maceration was carried out sequentially, beginning with ethyl acetate, followed by methanol. This approach was selected to capture a broader range of phytochemicals based on their polarity. The 2 L solvent volume per 300-400 g of plant material is based on other studies such as Rwegoshora et al. (2022). To improve clarity, we revised the sentence accordingly (lines 178-180):

*“For this process, approximately 300 g or 400 g of each plant part (depending on its availability and density) was packed in amber bottles and extracted sequentially with 2 L of solvent, beginning with EtOAc and followed by MeOH”.*

Line 151. Please move the comma here to behind the word “times” rather than behind the word “thereby” for grammatical correctness. “…was repeated three times, thereby ensuring maximum extraction”.

**Response:** We appreciate you pointing out the punctuation issue. We have revised the sentence for grammatical correctness as follows on lines 181-183:

*“The extraction process for each solvent lasted for 72 h at room temperature, and each extraction was repeated three times, thereby ensuring maximum extraction”.*

Line 151-154. Was the extract passed through a Whatman number 1 filter paper? What method was “used” pertaining to the filter paper? Please remove the comma after the word ‘was’, it should read as “was concentrated”, then please add the word “then” before the word ‘weighed’, it should read as “…remove moisture, then weighed and stored at…” Also, why negative 4 °C as a temperature? 4 degrees Celsius and negative 20 degrees Celsius are two common temperatures, but was there a reason for this specific temperature?

**Response:** Thank you, reviewer, for your time and detailed feedback on Lines 151-154 of the original manuscript. The revised sentence is on Lines 183-186 as follows:

*“The extracts were filtered using Whatman Nº 1 filter paper under gravity-assisted filtration. The resulting filtrate was then concentrated using a rotary evaporator (BUCHI), air-dried to evaporate the remaining solvent, placed in the desiccator to remove remaining moisture, then weighed and stored at - 20 ºC for further analysis."*

We acknowledge that 4 °C and – 20 °C are two common temperatures reported in the literature. The initially reported temperature of – 4 °C was an oversight due to the absence of a temperature indicator in the storage cooler. Subsequent measurement with a calibrated thermometer confirmed that the actual storage temperature was – 20 °C, typically required for long-term preservation.

Line 154. What exactly is a “crude extract” following a cold maceration? What was the original plant matter that formed the 24 crude extracts (stems, roots, etc.)? How many of the 24 samples were processed from each individual species of plant?

**Response:** We thank the reviewer for the thoughtful question concerning 154 in our original manuscript. A crude extract following a cold maceration is the initial product of the extraction process, consisting of a complex mixture of compounds derived from the original material, in which the raw material is soaked in a solvent. The original plants materials that formed the 24 crude extracts was root bark, root wood, stem bark, stem wood and leaves (Table 2 in the manuscript). From *A. stenophylla* were processed 4 samples (root bark, root wood, stem and leaves); *S. petersianus* 3 samples (roots, stems and leaves), and *S. glaucescens* 5 samples (root wood, root bark, stem bark, stem wood and leaves). Since each sample was sequentially extracted using two different solvents (ethyl acetate and methanol), the resulting extracts are designated as fractions or crude extracts. The revised sentence is on Lines 186-188 and reads as follows:

*“A total of 24 crude extracts (ethyl acetate and methanol) were obtained, and their yields were calculated using the formula provided below, and the yield values can be viewed within Table 3 of the results section.”*

Line 154. Please correct this sentence for grammar. A suggestion reads as follows, “24 crude extracts were obtained, their yields were calculated using the formula below, and the yield values can be viewed within Table 2 of the results section.”

**Response:** Thank you for your helpful suggestion regarding the phrasing in Line 154 in the original manuscript. Accordingly, we have revised with a slight refinement for flow and formal tone. Revised sentence now on lines 186-188 reads as follows:

*“A total of 24 crude extracts (ethyl acetate and methanol) were obtained, their yields were calculated using the formula provided below, and the yield values can be viewed within Table 3 of the results section”.*

Line 161. Could an image/photograph of the container used to hatch the eggs be included? It is difficult to visualize with only what is written and would benefit future readers if they intend to follow a similar method described here.

**Response:** Thank you for the valuable suggestion. We have now added a photograph of the hatching container as **Fig 1** in the revised manuscript to better illustrate the setup for future readers.

Fig. 1: Hatching container used for egg incubation and larval development

Line 163. What type of sea salt was used for the artificial seawater? Could a vendor and item/catalog number be provided here?

**Response**: Thank you for pointing this out. In this study, we used crude artificial sea salt prepared in the laboratory. The revised sentence, now on lines 200-201 reads as follows:

*“The crude artificial sea salt used was produced in the Laboratory by evaporating seawater collected from the Indian Ocean in Dar es Salaam, as detailed in Appendix 1”*

Line 164-165. When the container is described above, could the authors also include the lighting setup? It is unclear if this is a different container, where the lights are positioned, and why the lights were necessary for the hatching of larvae. If larvae were attracted to light at 24 hours, what purpose did collecting them at 48 hours serve?

**Response**: Thank you for your insightful comment regarding the lighting setup and larval collection timeline. We have now updated the manuscript to include a description of the lighting conditions, the positioning of the light source, and the rationale for the 48-hour collection time point. Specifically, we clarify that the same container described earlier was used throughout the hatching and collection process. A white LED source was positioned approximately 10 cm above the water surface to stimulate daylight conditions and encourage positive phototaxis in the larvae. Although some larvae became motile and photoreactive at 24 h, collection was performed at 48 h to allow complete hatching and to ensure the inclusion of all viable nauplii for testing. The revised sentence on lines 202-209 reads as follows:

*“The smaller compartment was illuminated using a white LED lamp positioned approximately 10 cm above the water surface to stimulate phototactic movement and support larvae hatching. After 24 h, hatched A. salina larvae migrated toward the smaller, illuminated compartment due to the phototactic behaviour.The larvae were collected from the lighted side, which was separated from the eggshells by a divider, after 48 h, using a 9-inch disposable pipette for use in the bioassay. The collection time allowed for complete hatching ensured adequate, viable, uniformly developed nauplii for the toxicity assay”.*

Line 159 and 167. Please list out the publications you are citing in these places and consistently throughout the manuscript as Last Name, et al. followed by the bracketed numbered citations [XX]. For example (line 158-159), “The toxicity screening of all extracts was carried out by the BSLT method according to Credo et al. and Meyer et al. [18,37] with slight modifications”.

**Response**: Thank you, reviewer, for your helpful suggestion regarding citation consistency. We have revised the sentence on lines 213-214 and reads as follows:

*“The bioassay was conducted according to the procedures described by Credo et al. and Meyer et al. [20,43] with slight modifications”.*

Line 166. “Test sample preparation and implementation”. This section seems to be missing many details, and much clarification to describe to the reader the protocol and experimental process which was performed. Please elaborate. Moreover, the authors should heavily focus on grammar as sentences like “In each well plate were added 10 larvae of A. salina 2 days old, test sample and the total volume of 3 ml was completed with artificial seawater” lower the quality of writing and make it difficult for the reader to understand the true impact of the methodology employed

**Response**: We appreciate the reviewer’s observation concerning the need for additional clarity in the Test sample preparation and implementation section. We have substantially revised this section. The revised information is on lines 214-233 and reads as follows:

*“A total of 40 mg of each extract was accurately weighed, and dissolved in 1 mL of 1% dimethyl sulfoxide (DMSO) to prepare the stock solution. From this stock, a series of working solutions was prepared by dilution with artificial seawater to achieve final concentrations of 360, 240, 180, 80, 40, and 20 µg/mL, which were used to treat A. salina larvae.”*

*“Ten A. salina larvae 2 days old, were transferred into each well of a transparent, flat-bottomed 24-well plate (untreated), followed by the addition of 3 mL per well of the respective test sample. The experimental groups included the test samples, a negative control consisting of 1% DMSO in artificial seawater and a positive control, the methanolic extract of Catharanthus roseus. The A. salina larvae in the experimental group were exposed to the test samples at the specified concentration. The control group received only 1% DMSO artificial seawater, while the positive control group was tested with C. roseus extract, known for its cytotoxic properties [46]. The experiment was conducted under consistent environmental conditions, such as a clean, well-ventilated area maintained at approximately 25 ºC under continuous ambient light (~1000 lux), to simulate natural conditions and support the normal behaviour of A. salina larvae during the 24-hour exposure period. Each concentration was tested in triplicate (n = 3 wells per concentration), providing a total of 30 larvae per concentration”.*

*“After 24 hours of exposure, the number of live and dead larvae was recorded, and the mortality rate was calculated using the following equation. Larvae were considered dead if they showed no movement during observation under a magnifying lens, even after a gentle agitation”.*

Line 170-171. What type of plates were used here (6-well, 12-well, etc. ; treated vs. untreated; clear vs. opaque)? Please provide details. Was 3 mL the total volume of seawater the A. salina were added to? This sentence needs to be reworded with a focus on proper grammar, it is illegible as is.

**Response:** Thank you, reviewer, for pointing this out. We have clarified the type and characteristics of the plates used and reworded the sentence for clarity. The revised sentence now on lines 219-221 reads as follows:

*“Ten A. salina larvae 2 days old, were transferred into each well of a transparent, flat-bottomed 24-well plate (untreated), followed by the addition of 3 mL per well of the respective test sample”.*

Line 172. How were different concentrations of sample achieved? Where did these come from – A. salina larvae in 3 mL seawater or 5 mL extract stock solutions? This sentence being placed directly after the experimental setup of brine shrimp in seawater within welled plates does not make logical sense, what process was performed to achieve these concentrations and why are there so many different concentrations (360, 240, 180, 80, 40, 20 ug/mL)? Please provide more details.

**Response:** Thank you for your careful reading and thoughtful comment. The different concentrations were prepared by serial dilution from the stock solution. Thus, the revised sentence in the manuscript now on lines 214-218 reads as follow:

*“A total of 40 mg of each extract was accurately weighed, and dissolved in 1 mL of 1% dimethyl sulfoxide (DMSO) to prepare the stock solution. From this stock, a series of working solutions was prepared by dilution with artificial seawater to achieve final concentrations of 360, 240, 180, 80, 40, and 20 µg/mL, which were used to treat A. salina larvae”.*

A range of concentrations was tested to assess dose-dependent toxicity and determine the LC₅₀ values for each extract. This range ensures the detection of both sublethal and lethal effects, allowing for a comparison of toxicity across samples.

Line 173. Please define “sufficient light”. What is the purpose of placing the experiment plate within “sufficient light” for 24 hours?

**Response:** Thank you for your valuable comment. By “sufficient light”, we referred to continuous (~1000 lux), which simulates natural daylight conditions. The purpose of maintaining this lighting environment for 24 hours exposure period is to support the normal physiological behaviour and metabolic activity of *A. salina* larvae, which are phototropic and require light to remain active and suspended in the water. The updated sentence in the manuscript, now on lines 226-228, reads as follows:

*“The experiment was conducted under consistent environmental conditions, such as a clean, well-ventilated area maintained at approximately 25 ºC under continuous ambient light (~1000 lux), to simulate natural conditions and support the normal behaviour of A. salina larvae during the 24-hour exposure period.”*

Line 174. Please provide more details about the standard solution and its composition (volumes, etc.). Was this used as a negative control? Were any dead A. salina found within the standard solution throughout the experiment? Moreover, were positive controls considered for this experiment, such as another natural product that is currently being used as a cancer treatment with an LC50 that could be compared to the three plant species in focus here?

**Response**: Thank you, reviewer, for your insightful questions and for highlighting the need for further clarification. In this study, the standard solution refers to the negative control, which was prepared by dissolving 1 mL of dimethyl sulfoxide (DMSO) in 99 mL of artificial seawater, resulting in a 1% DMSO solution with a total volume of 100 mL. This solution was used to ensure that the solvent alone did not cause mortality or interfere with the results. Throughout the experiment, no significant mortality was observed in the negative control group, confirming that 1% DMSO was non-toxic to the larvae under the test conditions. For positive control, we used a methanolic extract of *Catharanthus roseus,* a well-documented medicinal plant with known cytotoxic activity and relevance in cancer studies. This choice allowed us to compare the LC_50_ of the test extracts against a biologically active natural product with an established profile. We have revised the manuscript accordingly to include these details. The revised sentence now on lines 221-222 reads as follows.

*“The experimental groups included the test samples, a negative control consisting of 1% DMSO in artificial seawater and a positive control, the methanolic extract of Catharanthus roseus.”*

Line 182-183. Could brief additional details be added here to describe what the probit value is for readers that are unfamiliar? How is that value calculated from the percentage of A. salina deaths?

**Response**: We appreciate your helpful suggestion. We have now added a short description of the concept and process used to derive it from mortality data. The mortality rate of A. salina was converted into probit values, which reflect the standardised deviation of a given response from the mean of a normal distribution. This transformation enables linear analysis of dose-response relationships. Thus, probit values were obtained from the percentage mortality using Finney’s probit table and plotted against the logarithm (base 10) of extract concentrations. The median lethal concentration (LC_50_) was calculated using linear regression analysis in Microsoft Excel (version 2013) with a 95% confidence interval. The LC_50_ was derived from the linear regression equation $(y=ax+b)$ based on the point at which probit =5.

In the present version of the manuscript, we recalculate the LC_50_ values using Python as explained on lines 235-249 (statistical analysis section):

*“The median lethal concentration (LC_50_) values of A. salina (Brine shrimp) in both control and treatment groups were estimated using the four-parameter Hill equation [47].*

$$Y=Bottom+ \frac{(Top - Bottom)}{{(1 + (X / IC50)}^{^HillSlope}}$$

*Where:*

*Y represents the percentage mortality*

*X represents concentration in μg/mL.*

*Bottom and Top represent the minimum and maximum response values, respectively, and IC_50_ corresponds to the concentration causing 50% mortality.*

$$R^{2}=1-\frac{{SS}_{res}}{{SS}_{tot}}+\ldots$$

*Data handling and visualization were performed in Python 3 x using NumPy, SciPy, Pandas, and Matplotlib libraries”.*

Line 186. Please add in “Credo et al.” prior to the citation. Consider moving the graph to the results section rather than keeping this in the methods. Moreover, please define within the text what Y means and how that differs from the Predicted Y. Is there a linear regression for each of the samples that were listed within Table 2? It is confusing to me as a reader, as I am unfamiliar with LC50 calculations, why a linear regression is being mentioned with minimal context for how these two analyses are coupled.

**Response**: We sincerely thank the reviewer for the insightful comments and suggestions. In response, we have reanalysed the data using Python, which resulted in an updated statistical approach and visualisation. Consequently, the previous linear regression and reference to Y and Predicted Y were removed as these elements are no longer applicable in the revised analysis. The new Python analysis provides improved accuracy and reproducibility, generating a new graph that is now included in the Results section. Corresponding updates have been made to the **Methods** and **Results** sections to describe the revised analytical procedure and reflect the updated figure.

Line 192-197. Thank you for describing the two different toxicity indexes as that is informative to the reader. How were these indexes used within study to interpret the results?

**Response:** We thank the reviewer for this positive comment. The two toxicity indices were used to guide the interpretation of LC₅₀ values obtained from the bioassay. These indexes intersect at the LC₅₀ threshold of 1000 µg/mL, which is generally accepted as the cut-off for non-toxic classification. Clarkson’s toxicity index was applied to categorize the extracts into four levels: non-toxic (LC₅₀ > 1000 µg/mL), low toxicity (LC₅₀ = 500–1000 µg/mL), moderate toxicity (LC₅₀ = 100–500 µg/mL), and high toxicity (LC₅₀ < 100 µg/mL). This approach enabled a consistent and comparative evaluation of the toxicological profiles of the tested extracts.

# **RESULTS**

Line 202-205. The way the methods section “extraction” reads makes it seem as though the protocol utilized to create plant extracts is a combination of EtOAc with MeOH, but within the first paragraph of the results section, it seems that there were a set of extracts that were only treated with EtOAc. Can the authors provide clarification, and more detail about the process used to create extracts and describe each of the conditions assessed by the BSLT test?

**Response**: We thank the reviewer for this important observation. We have revised the Methods section to differentiate the extraction procedures used for each plant material. The updated text in the manuscript is in the Materials and Methods section on lines 173-175.

*“Each harvested plant part (Table 2) was subjected to total and sequential solvent extraction using cold maceration, starting with ethyl acetate (EtOAc) to extract low to medium polarity compounds, followed by methanol (MeOH) to obtain more polar constituents.”*

We also clarified the conditions under which each extract was evaluated using the BSLT the revised information, now on lines 211-214, reads as follows:

*“Each EtOAc and MeOH extract obtained through sequential extraction was independently evaluated under the same assay conditions using the BSLT to determine their respective LC_50_ values. The bioassay was conducted according to the procedures described by Pohan et al. and Meyer et al. [35,43] with slight modifications.”*

Line 205-207. What were the mortality values and LC50 values for each of the plants/parts? There is no mention of a figure or a table to reference within this sentence. Coming back to this, the second paragraph starting at line 208 reads in a more detailed manner and I see references Table 2. My suggestion would be to move the last two sentences (lines 203-207) to an appropriate place in the discussion section, as this text reads more as discussion than results.

**Response**: We thank the reviewer for the insightful comment. Accordingly, we have moved these sentences to the discussion section and revised the results section to include a clear reference to Table 3, where mortality and LC₅₀ values for each extract are presented. Concerning the mortality values and LC_50_ values for each of the plants/parts. The updated text, now on lines 330-333, reads as follows:

*“In general, at the tested concentrations (360, 240, 180, 80, 40 and 20 μg/ml), the EtOAc extracts demonstrated higher toxicity against A. salina larvae, as indicated by their greater average mortality rates and lower LC_50_ values (Table 3). In contrast, extracts from S. petersianus exhibited slightly higher LC_50_ values compared to those of the other two ant species”.*

Table 2. How many replicates are being averaged for each of the “average number of dead larvae”? If 10 fish are placed per well, then to get an average value with a decimal place there must be multiple values that are being averaged. Please add and describe the justification for the sample size as well as number of experiments performed within the results section.

**Response**: We appreciate the reviewer's helpful comment. We clarify that for each concentration, three replicates were performed (n = 3 wells per concentration), with 10 *A. salina* larvae per well. The average number of dead larvae per concentration was calculated based on these three replicates, which explains the appearance of decimal values in Table 3. This sample size (3 wells with 10 larvae per concentration) follows established protocols in the brine shrimp lethality assay literature. It is generally accepted as sufficient to ensure reliable and reproducible LC₅₀ estimations within a single experimental run. To address this, we have revised the Materials and Methods section to clearly state the number of replicates (lines 229-230), the number of larvae per well, and the justification for the sample size. We have also briefly clarified this point in the Results section when referring now to Table 3.

*“Each concentration was tested in triplicate (n = 3 wells per concentration), providing a total of 30 larvae per concentration”.*

Table 2. Why, if a concentration series of 360, 240, 180, 80, 40, and 20 ug/ml was created, were concentrations of 4000, 6000, 200, 8000, and 10000 ug/ml tested and recorded as final values in table 2? The context of where the concentration series comes from and why it was made is significantly lacking, moreover, how it directly relates to the reported results is unclear.

**Response**: We thank the reviewer for this insightful observation and appreciate the opportunity to clarify. The concentrations, such as 4000, 6000, 8000, and 10000 µg/mL, represent the **stock solutions** prepared for each extract. From these higher stock concentrations, we prepared the **working solutions** through dilution to achieve the test concentrations ranging from **20 to 360 µg/mL**, which were used in the brine shrimp lethality assays. The **final concentrations tested in the experiment and reported now in Table 3** are **20, 40, 80, 180, 240, and 360 µg/mL.** The mention of higher stock concentrations in the previous version of the manuscript may have confused.

Table 3. Please also add some description for how the % yield/g values are being reported. It does not make sense how a concentrate of, say, leaves of A. stenophylla EtOAc, would have a 12.42 % yield per gram with a (standard deviation, standard error? Please clarify) of 235 as one example.

**Response**: We thank the reviewer for this helpful comment and the opportunity to correct and clarify. The % yield reported in Table 3 was calculated as the weight of the dried extract relative to the initial weight of the plant material used for extraction. The extraction yield was expressed as a percentage (% w/w) and represents a single measurement obtained from the extraction process. The "± 235" was mistakenly included in the table and does not represent standard deviation or standard error. This was an error in data presentation, and we have now removed all such notations. In the revised version of Table 3, only the single % yield value is reported, with no error terms, to accurately reflect the experimental procedure.

Figure 1. I was expecting to read something within the results that relates back to the linear regression analysis explained within the methods. It struck me as odd that a linear regression graph/figure was included within the methods section titled “statistical analysis”, rather than a simple explanation that a linear regression analysis was performed that is used for LC50 calculations.

**Response**: We appreciate the reviewer’s helpful observation. In response, we have removed the linear regression figure from the Methods section. The LC₅₀ values were recalculated using Python, and the results are now illustrated in Fig 2 within results section.

**Fig 2. LC_50_ values of plant extracts compared to the positive control determined by BSLT assay**

# **Discussion**

Line 228-231. Please split this sentence up.

**Response**: Thank you for this helpful suggestion. We have revised the sentence as recommended and split it into two clearer and more concise sentences in the revised manuscript. The updated text, now on lines 303-305, reads as follows:

*“The BSLT method was used for screening the toxicity of the extracts from the species under study because this method has been shown to correlate positively with cytotoxic effect on cancer cells.”*

Line 234. How do you know these bioactive compounds are phenolic, alkaloids, etc.? Were confirmation tests done to assess these compounds or is this claim supported by other research, in that the LC50 values being relatively low provide evidence for this?

**Response**: Thank you for your comment. We did not conduct specific phytochemical confirmation tests for these fractions. However, the inference regarding the presence of phenolic compounds, alkaloids, and other secondary metabolites is based on previously published studies reporting similar LC₅₀ values and biological activity patterns for plant extracts containing such compounds. This has been clarified and supported with references in the revised manuscript on lines 310-316 as follows:

*“The extracts of A. stenophylla and S. glaucescens exhibited relatively low LC_50_ values (LC_50_ < 100 μg/mL), indicating strong cytotoxic activity [49]. These findings suggest the presence of potent bioactive compounds with various pharmacological properties, including potential anticancer activity [37,55]. Although specific compound confirmation was not conducted in this study, the observed cytotoxic profiles are consistent with previous reports linking such activity to secondary metabolites, phenolic compounds, alkaloids, terpenoids, acetogenins, terpenes, tannins and other secondary metabolites in related studies.”*

Line 242-245. It is counterintuitive to state that based on results, extracts show promise for isolation and purification of extracts. I believe the authors are trying to describe that the different parts of the species, based on their LC50 values, show promise as possible cancer treatments/have anticancer properties, which is also supported by the research mentioned within line 246. Please consider the message intended for the reader, and how it relates to the specific experiments done, in addition to how this study may be expanding the field of cancer treatment in a novel way.

**Response**: Thank you for this valuable comment. We have revised the statement to better reflect the intended message. The revised sentence, now on lines 347-350, reads as follows:

*“In this study, certain plant parts exhibited notable cytotoxic activity based on their LC_50_ values, suggesting the presence of bioactive constituents that warrant further isolation, characterization and evaluation for potential anticancer applications.”*

Line 250. If information is limited for this plant, what information is available? Please include. If there is a knowledge gap with this species of plant, where minimal information is known about it then stating that alongside the possibility of it being used for cancer treatment could shape that knowledge gap into productive future research

**Response:** Thank you for this valuable suggestion. The revised text on the manuscript on lines 356-364 reads as follow:

*“Although scientific information on S. petersianus remains limited, a few studies have documented its traditional use as an arrow poison by several African communities, including the Zulu, attributed to its content of potent cardiac glycosides. Additionally, it has been employed in ritualistic and protective practices aimed and averting harm. However, its cytotoxic and pharmacological properties, as well as its phytochemical profile, remain largely unexplored. This study contributes to addressing this knowledge gap by demonstrating the cytotoxic effects of S. petersianus extracts, suggesting its potential as a promising candidate for future anticancer research. Notably, other species within the Strophanthus genus have been reported to exhibit antimicrobial, wound-healing, antioxidant, analgesic, and anticarcinogenic properties”.*

# **Conclusion**

Please expand, this current conclusion does not adequately summarize the entire study, explain key takeaways to the reader from what was found, nor does it mention limitations and future direction. This could be expanded upon with breadth of information rather than sheer length and allow for a more satisfactory closing for whether the hypothesis was approved or rejected.

Within the introduction, there is a mention that more cancer treatments with minimal adverse effects are needed, due to the projected growth of cancer in the human population. I find this to be a fascinating take and the investigation into the toxicity of medicinal plants an interesting approach to address this “need” within the scientific field/cancer research field. As a suggestion, mention how the results of this study help address that research need, which is described well in the introduction, here in the conclusion, so that everything throughout the manuscript comes full circle.

**Response**: Thank you for your thoughtful and constructive feedback. we have significantly revised the conclusion to:

*“This study demonstrated that extracts from A. stenophylla, S. petersianus and S. glaucescens exhibit significant cytotoxic activity, supporting their traditional medicinal use and validating our hypothesis that this plant contains bioactive compounds with potential anticancer properties. Among the tested extracts, the root wood methanolic fraction from A. stenophylla exhibited the highest toxicity, indicating the presence of potent cytotoxic constituents extractable by this solvent”.*

*“Although the use of C. roseus as a positive control and 1%DMSO as a negative control ensured the reliability of the results, several limitations must be acknowledged. This includes a limited sample size of the plant materials, a limited number of test organisms per well, a lack of detailed mechanistic studies, for instance, apoptosis, oxidative stress, and the absence of in vivo validation. These factors may constrain the broader applicability of the findings”.*

*“Nonetheless, contribute to ongoing efforts to discover new anticancer agents with fewer adverse effects, as highlighted in the introduction. The observed cytotoxic provides preliminary evidence that this species could serve as a promising source of lead compounds. Future studies should focus on bioassay-guided isolation of active constituents, detailed phytochemical profiling, mechanistic studies and evaluation in more complex biological models to further assess efficacy and safety”.*

*“Considering the increasing global burden of cancer and the urgent need for safer, more effective treatments, this study represents a step forward in the scientific exploration of traditional medicinal plants as potential contributors to cancer drug discovery”.*

We are confident that these revisions have substantially improved the manuscript and that it is now more suitable for publication in PLOS ONE. We are grateful for your time and continued consideration and look forward to your decision.

Yours sincerely.

Roberto Luis Nhamussua
